# Phenotypic characteristics and prognosis of inpatients with COVID-19 and diabetes: the CORONADO study

**DOI:** 10.1007/s00125-020-05180-x

**Published:** 2020-05-29

**Authors:** Bertrand Cariou, Samy Hadjadj, Matthieu Wargny, Matthieu Pichelin, Abdallah Al-Salameh, Ingrid Allix, Coralie Amadou, Gwénaëlle Arnault, Florence Baudoux, Bernard Bauduceau, Sophie Borot, Muriel Bourgeon-Ghittori, Olivier Bourron, David Boutoille, France Cazenave-Roblot, Claude Chaumeil, Emmanuel Cosson, Sandrine Coudol, Patrice Darmon, Emmanuel Disse, Amélie Ducet-Boiffard, Bénédicte Gaborit, Michael Joubert, Véronique Kerlan, Bruno Laviolle, Lucien Marchand, Laurent Meyer, Louis Potier, Gaëtan Prevost, Jean-Pierre Riveline, René Robert, Pierre-Jean Saulnier, Ariane Sultan, Jean-François Thébaut, Charles Thivolet, Blandine Tramunt, Camille Vatier, Ronan Roussel, Jean-François Gautier, Pierre Gourdy, for the CORONADO investigators

**Affiliations:** 1 Département d’Endocrinologie, Diabétologie et Nutrition, l’institut du thorax, Inserm, CNRS, UNIV Nantes, CHU Nantes, Hôpital Guillaume et René Laennec, 44093 Nantes Cedex 01, France; 2 CIC-EC 1413, Clinique des Données, CHU Nantes, Nantes, France; 3 Département d’Endocrinologie, Diabétologie et Nutrition, CHU Amiens, PeriToxUMR_I 01, Université de Picardie, Amiens, France; 4 Département d’Endocrinologie, Diabétologie, Nutrition, CHU de Angers, Angers, France; 5 Département de Diabétologie, Centre Hospitalier Sud Francilien, Corbeil Essonne, France; 6 Département d’Endocrinologie, Diabétologie et Maladies Métaboliques, Centre Hospitalier Bretagne Atlantique, Vannes, France; 7 Clinique d’Endocrinologique Marc-Linquette, Hôpital Claude-Huriez, CHRU de Lille, Lille, France; 8 Département de Diabétologie, H.I.A. Begin, Saint Mandé, France; 9 Fondation Francophone pour la Recherche sur le Diabète (FFRD), Paris, France; 10 Département d’Endocrinologie, Diabétologie et Nutrition, CHU de Besançon, Besançon, France; 11 Département d’Endocrinologie, Diabétologie et Nutrition, Assistance Publique Hôpitaux de Paris, Université Paris Saclay, Hôpital Antoine Béclère, Clamart, Hôpital Bicêtre, Le Kremlin Bicêtre, France; 12 Sorbonne Université, Assistance Publique Hôpitaux de Paris, Département de Diabétologie, CHU La Pitié Salpêtrière-Charles Foix, Inserm, UMR_S 1138, Centre de Recherche des Cordeliers, Paris 06, Institute of Cardiometabolism and Nutrition ICAN, Paris, France; 13 Département des Maladies Infectieuses et Tropicales, CHU Nantes, Nantes, France; 14 Département des Maladies Infectieuses et Tropicales, CHU de Poitiers, INSERM U1070, Poitiers, France; 15 Société de Pathologie Infectieuse de langue Française (SPILF), Paris, France; 16 Fédération Française des Diabétiques (FFD), Paris, France; 17 Assistance Publique Hôpitaux de Paris, Hôpital Avicenne, Université Paris 13, Sorbonne Paris Cité, Département d’Endocrinologie, Diabétologie et Nutrition, CRNH-IdF, CINFO, Bobigny, France; 18 Université Paris 13, Sorbonne Paris Cité, UMR U557 Inserm / U11125 INRAE / CNAM / Université Paris13, Unité de Recherche Epidémiologique Nutritionnelle, Bobigny, France; 19 Département d’Endocrinologie et de Diabétologie, Hôpital de la Conception, Assistance Publique Hôpitaux de Marseille, Marseille, France; 20 Département d’Endocrinologie, Diabétologie et Nutrition, Hospices Civils de Lyon, CarMeN Laboratory, Inserm 1060, Lyon, France, Université Claude Bernard Lyon 1, Lyon, France; 21 Département d’Endocrinologie et de Diabétologie, Centre Hospitalier Départemental de Vendée, La Roche sur Yon, France; 22 Département d’Endocrinologie et de Diabétologie, Hôpital Nord, Assistance Publique Hôpitaux de Marseille, Marseille, France; 23 Département de Diabétologie, CHU de Caen, Caen, France; 24 Département d’Endocrinologie, CHU de Brest, EA 3878 GETBO, Brest, France; 25 Université de Rennes, CHU Rennes, Inserm, CIC 1414 (Centre d’Investigation Clinique de Rennes), Rennes, France; 26 Département d’Endocrinologie et de Diabétologie, Centre Hospitalier St. Joseph - St. Luc, Lyon, France; 27 Département d’Endocrinologie, Diabétologie et Nutrition, Hôpitaux Universitaires de Strasbourg, Strasbourg, France; 28 Département d’Endocrinologie, Diabétologie et Nutrition, Hôpital Bichat, Assistance Publique Hôpitaux de Paris, Centre de Recherche des Cordeliers, Inserm, U-1138, Université de Paris, Paris, France; 29 Département d’Endocrinologie, Diabétologie et Maladies Métaboliques, CHU de Rouen, Université de Rouen, Rouen, France; 30 Département Diabète et Endocrinologie, Hôpital Lariboisière, Assistance Publique Hôpitaux de Paris, Paris, France; 31 Paris Diderot–Paris VII Université, Paris, France; 32 Inserm UMRS 1138, Université Paris Diderot–Paris VII, Sorbonne Paris Cité, Paris, France; 33 Université de Poitiers, CIC Inserm 1402, Poitiers, Médecine Intensive Réanimation, Poitiers, France; 34 Centre d’Investigation Clinique CIC 1402, Université de Poitiers, Inserm, CHU de Poitiers, Poitiers, France; 35 Département d’Endocrinologie, Diabète, Nutrition et CIC Inserm 1411, CHU de Montpellier, Montpellier, France; 36 Centre du Diabète DIAB-eCARE, Hospices Civils de Lyon et Laboratoire CarMeN, Inserm, INRA, INSA, Université Claude Bernard Lyon 1, Lyon, France; 37 Société Francophone du Diabète (SFD), Paris, France; 38 Département d’Endocrinologie, Diabétologie et Nutrition, CHU Toulouse, Institut des Maladies Métaboliques et Cardiovasculaires, UMR1048 Inserm/UPS, Université de Toulouse, Toulouse, France; 39 Assistance Publique Hôpitaux de Paris, Saint-Antoine Hospital, Reference Center of Rare Diseases of Insulin Secretion and Insulin Sensitivity (PRISIS), Department of Endocrinology, Paris, France; 40 Sorbonne University, Inserm UMRS 938, Saint-Antoine Research Center, Paris, France

**Keywords:** BMI, COVID-19, Death, Diabetes, HbA_1c_, Hypertension, Mechanical ventilation

## Abstract

**Aims/hypothesis:**

Coronavirus disease-2019 (COVID-19) is a life-threatening infection caused by the severe acute respiratory syndrome coronavirus-2 (SARS-CoV-2) virus. Diabetes has rapidly emerged as a major comorbidity for COVID-19 severity. However, the phenotypic characteristics of diabetes in COVID-19 patients are unknown.

**Methods:**

We conducted a nationwide multicentre observational study in people with diabetes hospitalised for COVID-19 in 53 French centres in the period 10–31 March 2020. The primary outcome combined tracheal intubation for mechanical ventilation and/or death within 7 days of admission. Age- and sex-adjusted multivariable logistic regressions were performed to assess the prognostic value of clinical and biological features with the endpoint. ORs are reported for a 1 SD increase after standardisation.

**Results:**

The current analysis focused on 1317 participants: 64.9% men, mean age 69.8 ± 13.0 years, median BMI 28.4 (25th–75th percentile: 25.0–32.7) kg/m^2^; with a predominance of type 2 diabetes (88.5%). Microvascular and macrovascular diabetic complications were found in 46.8% and 40.8% of cases, respectively. The primary outcome was encountered in 29.0% (95% CI 26.6, 31.5) of participants, while 10.6% (9.0, 12.4) died and 18.0% (16.0, 20.2) were discharged on day 7. In univariate analysis, characteristics prior to admission significantly associated with the primary outcome were sex, BMI and previous treatment with renin–angiotensin–aldosterone system (RAAS) blockers, but not age, type of diabetes, HbA_1c_, diabetic complications or glucose-lowering therapies. In multivariable analyses with covariates prior to admission, only BMI remained positively associated with the primary outcome (OR 1.28 [1.10, 1.47]). On admission, dyspnoea (OR 2.10 [1.31, 3.35]), as well as lymphocyte count (OR 0.67 [0.50, 0.88]), C-reactive protein (OR 1.93 [1.43, 2.59]) and AST (OR 2.23 [1.70, 2.93]) levels were independent predictors of the primary outcome. Finally, age (OR 2.48 [1.74, 3.53]), treated obstructive sleep apnoea (OR 2.80 [1.46, 5.38]), and microvascular (OR 2.14 [1.16, 3.94]) and macrovascular complications (OR 2.54 [1.44, 4.50]) were independently associated with the risk of death on day 7.

**Conclusions/interpretations:**

In people with diabetes hospitalised for COVID-19, BMI, but not long-term glucose control, was positively and independently associated with tracheal intubation and/or death within 7 days.

**Trial registration:**

clinicaltrials.gov NCT04324736.
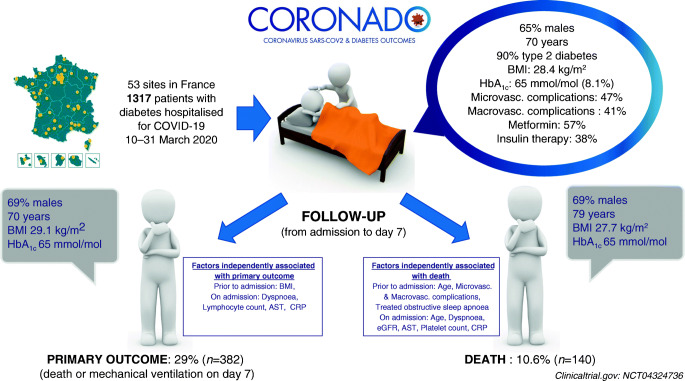

**Electronic supplementary material:**

The online version of this article (10.1007/s00125-020-05180-x) contains peer-reviewed but unedited supplementary material, which is available to authorised users.

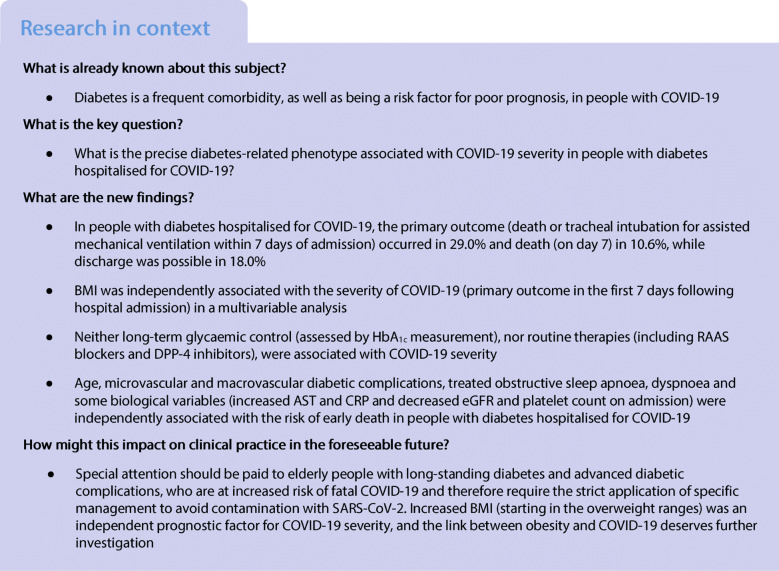



## Introduction

Since the first case in China in December 2019, the epidemic of coronavirus disease-2019 (COVID-19), a disease caused by the severe acute respiratory syndrome-coronavirus-2 (SARS-CoV-2) virus, rapidly spread worldwide and was declared a pandemic by the World Health Organization on 11 March 2020 [1, 2].

It is well known that people with diabetes have increased infection risk, especially for influenza and pneumonia [3, 4]. Moreover, diabetes was previously reported as a major risk factor for mortality in people infected with the 2009 H1N1 pandemic influenza and, more recently, with the Middle East respiratory syndrome-related coronavirus (MERS-CoV) [5, 6]. Epidemiological studies have quickly and consistently pointed out diabetes as one of the major comorbidities associated with COVID-19 and affecting its severity.

The prevalence of diabetes in patients with COVID-19 was first reported to range from 5% to 20% in Chinese studies, increasing with the severity of the disease [7]. More recently, Grasselli et al have reported a diabetes prevalence of 17% in patients admitted to intensive care units (ICUs) for severe COVID-19 infection in Lombardy, Italy [8]. Furthermore, the COVID-19-Associated Hospitalisation Surveillance Network (COVID-NET) reported a diabetes prevalence of 28.3% in hospitalised patients in the USA [9].

More importantly, all studies published so far have reported a two- to threefold higher prevalence of diabetes in patients in ICUs compared with those with less severe disease and an increased mortality in people with diabetes [10–14]. For instance, in a retrospective study from Wuhan, diabetes was present in 19% of 191 COVID-19 inpatients but its prevalence raised to 31% in deceased people compared with 14% in those who survived [12]. A recent meta-analysis further demonstrated that diabetes was associated with a more than doubled risk for ICU admission and a more than tripled risk for death [14].

In this context, patients with diabetes have been listed as people at higher risk for severe illness from COVID-19 by several health authorities and learned medical societies [15]. However, precise data regarding diabetes characteristics in hospitalised people with COVID-19 are still lacking. Moreover, the relationship between diabetes-related phenotypes and the severity of COVID-19 remains unknown. CORONADO (Coronavirus SARS-CoV-2 and Diabetes Outcomes) is a nationwide multicentre observational study that aims to identify the clinical and biological features associated with disease severity and mortality risk in people with diabetes hospitalised for COVID-19.

## Methods

### Study oversight

The CORONADO study was launched in all French hospitals volunteering to share data on hospitalised COVID-19 patients with diabetes. The study was sponsored by CHU (centre hospitalier universitaire) Nantes, designed in accordance with the declaration of Helsinki and conducted in accordance with French legislation with approval obtained from the local ethics committee (Institutional Review Board/Institutional Ethics Committee – GNEDS [groupe nantais d'éthique dans le domaine de la santé]; Ref. CORONADOV2), the CEREES (comité d'expertise pour les recherches, les études et les évaluations dans le domaine de la santé; n° INDS [institut national des données de santé]:1544730) and the CNIL (commission nationale de l'informatique et des libertés; DR-2020-155/920129). In light of the purely non-interventional design of this observational study and the emergency situation related to the COVID-19 pandemic, the CNIL and the GNEDS have repealed the systematic collection of written informed consent. They recommended that we collect an ‘oral non-opposition to participate’ as far as possible (in particular by publishing study information via posters in the hospitals). Living patients who were unable to give consent on admission all received information about their inclusion in the CORONADO study before discharge, and therefore had a clear and free choice to object to the use of their clinical data. Any patient declining to participate in the study or expressing his or her opposition to data collection from hospital information systems, even after hospital discharge, was excluded from the study.

### Study design and participants

The aim of the CORONADO study was to describe the phenotypic characteristics and prognosis of individuals admitted to hospital with COVID-19 between 10 March and 10 April 2020. Inclusion criteria were (1) hospitalisation in a dedicated COVID-19 unit with COVID-19 diagnosis confirmed biologically (by SARS-CoV-2 PCR test) and/or clinically/radiologically (i.e. as ground-glass opacity and/or crazy paving on chest computed tomography [CT] scan); (2) personal history of diabetes or newly diagnosed diabetes on admission (i.e. HbA_1c_ ≥48 mmol/mol [6.5%] during hospitalisation).

**Fig. 1:**
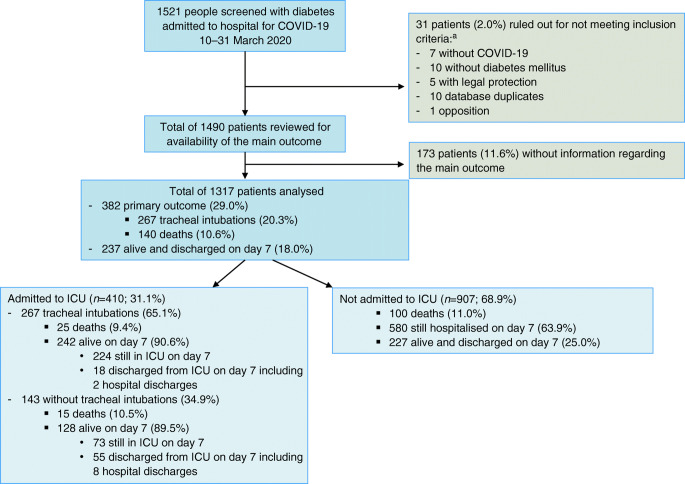
Study flowchart. ^a^Two patients ruled out for not meeting inclusion criteria were in two categories

Owing to the rapid recruitment rate and in order to make clinically relevant findings available as quickly as possible, the scientific committee, on 5 April 2020, suggested a premature database lock on 18 April 2020 for participants admitted in the period 10–31 March 2020, and continuation of recruitment with no further modification. A first set of analyses was performed in 1317 participants selected according to the following criteria: (1) meeting the eligibility criteria; (2) available information on the main outcome, recorded on day 7 following admission; (3) available data on age and sex (see flowchart in Fig. 1).

### Patient follow-up and clinical outcomes

The composite primary endpoint combined tracheal intubation for mechanical ventilation and death within 7 days of admission. Secondary outcomes included death on day 7, tracheal intubation on day 7, admission to ICUs and discharge on day 7. Participants discharged before day 7 were systematically contacted to check for the non-occurrence of these events on day 7.

### Data collection

Data collection was performed by clinical research associates and physicians in participating centres. They were instructed to systematically review the medical files of all COVID-19 inpatients, select those with diabetes, extract data from their medical files and, if needed, contact the patient’s general and/or specialist practitioners, regular pharmacist or biomedical laboratory. Collected data included clinical data (age, sex, ethnicity, BMI), classification of diabetes as noted in the medical file by the physician in charge of the patient, duration of diabetes, recent glycaemic control (i.e. two most recent HbA_1c_ levels determined before admission), microvascular and macrovascular complications and comorbidities. HbA_1c_ considered in the analysis was determined locally in the 7 days following admission or, if not available, was the result of a routine determination in the previous 6 months. Microvascular complications were defined as severe diabetic retinopathy (proliferative retinopathy and/or laser photocoagulation and/or clinically significant macular oedema requiring laser and/or intra-vitreal injections) and/or diabetic kidney disease (proteinuria [AER ≥300 mg/24 h; urinary albumin/creatinine ratio ≥300 mg/g; urinary albumin/creatinine ratio ≥30 mg/mmol creatinine; proteinuria ≥500 mg/24 h] and/or eGFR equal to or lower than 60 ml min^−1^ [1.73 m]^−2^, using the Chronic Kidney Disease Epidemiology Collaboration [CKD-EPI] formula) and/or history of diabetic foot ulcer. Macrovascular complications were defined as ischaemic heart disease (acute coronary syndrome and/or coronary artery revascularisation) and/or cerebrovascular disease (stroke and/or transient ischaemic attack) and/or peripheral artery disease (amputation owing to ischaemic disease and/or lower limb artery revascularisation). In addition, COVID-19-related clinical, radiological and biological characteristics were collected at admission as well as their clinical evolution during the hospital stay.

### Statistical analysis

Quantitative data were expressed as mean ± SD or median [25th –75th percentile]. Categorical variables were given as number (percentage) of participants. As prespecified in the protocol, the main objective of the study was descriptive. We calculated that a population size of 300 participants with an attrition of 20% for missing data, and a percentage of 16% of our main outcome, would give us a 95% CI equal to 11.7–21.1% using the Clopper–Pearson estimate.

Univariate logistic regression models were used to calculate OR associated with primary outcome or death on day 7. Natural log transformation was consistently considered in cases of skewed distribution, which applied to BMI and biological features.

Age- and sex-adjusted ORs for the primary outcome were plotted for BMI, HbA_1c_ and admission plasma glucose using degree 2 fractional polynomial approaches [16].

Multivariable logistic regression models were used to separately assess the association of the primary outcome and death on day 7 with clinical and biological features. A standardisation process was also applied using *z* scores for the purpose of direct comparison. In our initial statistical analysis plan, four covariates were systematically forced in the models: age, sex, BMI and HbA_1c_. However, since HbA_1c_ did not contribute to the risk of either the primary outcome or death on day 7, and owing to a significant number of missing data for HbA_1c_ and BMI, our multivariable models ultimately took only age and sex into account. Other variables were considered only if associated with the main outcome in univariate analysis (threshold: two-sided *p* value ≤0.10) and selected in the final model after a stepwise backward/forward selection process. In the event of obvious collinearity (such as alanine aminotransferase [ALT] with aspartate aminotransferase [AST], or white cell count with lymphocyte count), only the variable associated with the smaller p value was considered for multivariable analysis. In the final model, interactions were checked between all pairs of covariates.

We built two distinct multivariable models both separately for the main outcome and for the risk of death: (1) the first included covariates related to patient history prior to admission (chronic diabetes complications and other comorbidities) and routine medications; (2) the second included covariates related to medical presentation on admission, such as COVID-19 symptoms and biological determinations. This corresponds to the situation of a physician in an emergency room or department, assessing the prognosis of his/her patient.

All statistical tests were two-sided with a type 1 error set at 5%. All analyses were performed on available data, without imputation, and using statistical software R version 3.6.2 (https://cran.r-project.org/bin/windows/base/old/3.6.2/).

## Results

### Population and clinical outcomes

The present analysis focused on 1317 participants with diabetes and confirmed COVID-19 admitted to 53 French hospitals during the period 10–31 March 2020.

A total of 382 patients (29.0%; 95% CI 26.6, 31.5) met the primary outcome. Overall, 410 patients (31.1%; 95% CI 28.6, 33.7) were admitted to ICUs within 7 days of hospital admission, including 267 individuals who required tracheal intubation for mechanical ventilation (20.3%; 95% CI 18.1, 22.5). One hundred and forty deaths (10.6%; 95% CI 9.0, 12.4) were recorded on day 7. In contrast, 237 participants (18.0%; 95% CI 16.0, 20.2) were discharged on day 7 (see flowchart in Fig. 1).

### Demographic and diabetes-related characteristics

The clinical characteristics of the whole population are shown in Table 1. Mean (± SD) age was 69.8 ± 13.0 years and 64.9% were men. The classification of diabetes cases mainly included type 2 diabetes (88.5%), and less frequently type 1 diabetes (3.0%) or other aetiologies (5.4%). In addition, 3.1% of the participants were newly diagnosed with diabetes on admission (HbA_1c_ ≥48 mmol/mol [6.5%]). The median BMI was 28.4 (25th–75th percentile 25.0–32.7) kg/m^2^. The mean HbA_1c_ value was 65 ± 21 mmol/mol (8.1 ± 1.9%). A medical history of hypertension and dyslipidaemia were found in 77.2% and 51.0% of the participants, respectively. Microvascular and macrovascular complications were reported in 46.8% and 40.8% of individuals, respectively. Regarding routine glucose-lowering medications, 38.3% of the participants were on insulin therapy while 56.6% received metformin and 21.6% dipeptidyl peptidase 4 (DPP-4) inhibitors. Moreover, treatment with renin–angiotensin–aldosterone system (RAAS) blockers (ACE inhibitors and/or angiotensin II receptor blockers [ARBs] and/or mineralocorticoid-receptor antagonists [MRAs]) and statins was used by 57.1% and 47.6% of the participants, respectively.

**Table 1:** Clinical characteristics prior to admission of CORONADO participants, according to primary outcome (tracheal intubation and/or death within 7 days of admission), and death on day 7

Clinical features	Number of people with available data	All	Primary outcome (*n* = 382) OR (95% CI)	Death (*n* = 140) OR (95% CI)
Sex (female/male)	1317	462/1317 (35.1)	0.77 (0.60, 0.99)	0.80 (0.55, 1.17)
Age (years)^a^	1317	69.8 ± 13.0	1.00 (0.99, 1.01)	1.09 (1.07, 1.11)
Age class (years)	1317			
<55		159/1317 (12.1)	1	1
55–64		266/1317 (20.2)	0.58 (0.38, 0.90)	1.00 (0.23, 4.23)
65–74		394/1317 (29.9)	0.89 (0.60, 1.31)	3.22 (0.95, 10.1)
≥75		498/1317 (37.8)	0.85 (0.58, 1.24)	14.6 (4.56, 46.6)
Type of diabetes	1317			
Type 2		1166/1317 (88.5)	1	1
Type 1		39/1317 (3.0)	0.73 (0.35, 1.56)	0.44 (0.11, 1.86)
Other		71/1317 (5.4)	1.33 (0.80, 2.20)	1.50 (0.77, 2.93)
Diagnosed on admission		41/1317 (3.1)	0.79 (0.38, 1.63)	–
Ethnicity	1035			
EU		641/1035 (61.9)	1	1
MENA		196/1035 (18.9)	0.98 (0.69, 1.40)	0.87 (0.52, 1.47)
AC		174/1035 (16.8)	0.96 (0.66, 1.40)	0.78 (0.44, 1.37)
AS		24/1035 (2.3)	1.51 (0.65, 3.52)	–
BMI (kg/m^2^)^a^	1117	28.4 [25.0–32.7]	1.25 (1.09, 1.42)	0.95 (0.78, 1.16)
BMI class	1117			
<25 kg/m^2^		279/1117 (25)	1	1
25–29.9 kg/m^2^		410/1117 (36.7)	1.33 (0.93, 1.89)	0.70 (0.42, 1.16)
30–39.9 kg/m^2^		359/1117 (32.1)	1.71 (1.20, 2.43)	0.76 (0.45, 1.27)
≥40 kg/m^2^		69/1117 (6.2)	1.28 (0.70, 2.32)	0.74 (0.29, 1.84)
Diabetes duration (years)	772	13.6 ± 10.9	1.00 (0.98, 1.01)	1.01 (0.99, 1.04)
HbA_1c_ (mmol/mol)^a^	846	65.4 ± 21.2	0.99 (0.99, 1.00)	1.00 (0.99, 1.02)
HbA_1c_ (%)^a^	846	8.1 ± 1.9	0.94 (0.86, 1.03)	1.02 (0.87, 1.19)
HbA_1c_ (categories)	846			
<53 mmol/mol (7%)		245/846 (29.0)	1	1
53–63 mmol/mol (7–7.9%)		228/846 (27.0)	0.84 (0.55, 1.27)	1.55 (0.82, 2.93)
64–74 mmol/mol (8–8.9%)		164/846 (19.4)	0.92 (0.59, 1.45)	1.09 (0.52, 2.28)
≥75 mmol/mol (9%)		209/846 (24.7)	0.78 (0.51, 1.21)	0.84 (0.40, 1.75)
Hypertension	1299	1003/1299 (77.2)	1.23 (0.92, 1.65)	1.82 (1.11, 2.98)
Dyslipidaemia	1255	640/1255 (51.0)	1.07 (0.84, 1.37)	1.21 (0.84, 1.74)
Tobacco use	1029			
Never		582/1029 (56.6)	1	1
Former		390/1029 (37.9)	1.21 (0.91, 1.61)	1.00 (0.64, 1.57)
Current		57/1029 (5.5)	1.54 (0.87, 2.74)	1.20 (0.49, 2.93)
Long, term diabetes complications				
Microvascular complications	883	413/883 (46.8)	1.28 (0.94, 1.73)	5.25 (3.03, 9.10)
Severe diabetic retinopathy	954	66/954 (6.9)	1.22 (0.71, 2.11)	2.05 (1.03, 4.07)
Diabetic kidney disease	1066	355/1066 (33.3)	1.03 (0.78, 1.37)	3.19 (2.09, 4.87)
History of diabetic foot ulcer	1232	76/1232 (6.2)	0.67 (0.38, 1.18)	1.53 (0.79, 2.99)
Macrovascular complications	1189	485/1189 (40.8)	1.18 (0.91, 1.52)	3.58 (2.41, 5.31)
Ischaemic heart disease (ACS/CAR)	1251	336/1251 (26.9)	1.04 (0.79, 1.37)	2.65 (1.84, 3.82)
Cerebrovascular disease (stroke or TIA)	1267	163/1267 (12.9)	1.02 (0.71, 1.47)	2.19 (1.4, 3.42)
Peripheral artery disease (major amputation/LLAR)	1285	145/1285 (11.3)	0.91 (0.61, 1.34)	1.97 (1.23, 3.17)
Comorbidities				
Heart failure	1206	140/1206 (11.6)	0.78 (0.52, 1.17)	2.28 (1.42, 3.66)
NAFLD or liver cirrhosis	1107	119/1107 (10.7)	1.23 (0.81, 1.86)	0.70 (0.34, 1.41)
Active cancer	1282	194/1282 (15.1)	1.08 (0.77, 1.50)	1.55 (0.99, 2.42)
COPD	1278	133/1278 (10.4)	0.96 (0.64, 1.43)	1.36 (0.80, 2.32)
Treated OSA	1189	144/1189 (12.1)	1.44 (0.99, 2.08)	1.81 (1.12, 2.93)
Organ graft	1302	38/1302 (2.9)	1.14 (0.57, 2.28)	0.46 (0.11, 1.93)
End stage renal failure	831	60/831 (7.2)	0.66 (0.35, 1.27)	0.62 (0.24, 1.60)
Routine treatment before admission				
Metformin	1317	746/1317 (56.6)	0.95 (0.75, 1.21)	0.59 (0.42, 0.84)
Sulfonylurea/glinides	1317	367/1317 (27.9)	1.03 (0.79, 1.34)	0.74 (0.49, 1.13)
DPP-4 inhibitors	1317	285/1317 (21.6)	1.01 (0.75, 1.34)	0.85 (0.55, 1.32)
GLP1-RA	1317	123/1317 (9.3)	1.36 (0.92, 2.01)	0.64 (0.32, 1.29)
Insulin	1317	504/1317 (38.3)	1.01 (0.79, 1.29)	1.71 (1.20, 2.43)
Loop diuretics	1317	252/1317 (19.1)	1.10 (0.81, 1.48)	2.49 (1.70, 3.64)
Thiazide diuretics	1317	267/1317 (20.3)	1.08 (0.81, 1.45)	0.98 (0.63, 1.52)
Potassium-sparing diuretics	1317	59/1317 (4.5)	1.17 (0.67, 2.05)	1.77 (0.88, 3.58)
MRA	1317	53/1317 (4.0)	0.96 (0.52, 1.78)	2.03 (1.00, 4.13)
β-blockers	1317	442/1317 (33.6)	1.03 (0.80, 1.32)	1.84 (1.29, 2.62)
ACE inhibitors	1317	354/1317 (26.9)	1.17 (0.90, 1.52)	1.43 (0.99, 2.08)
ARBs	1317	389/1317 (29.5)	1.22 (0.94, 1.57)	1.15 (0.79, 1.67)
ARBs and/or ACE inhibitors	1317	737/1317 (56.0)	1.32 (1.03, 1.68)	1.58 (1.09, 2.28)
ARBs and/or ACE inhibitors and/or MRA	1317	752/1317 (57.1)	1.29 (1.01, 1.65)	1.67 (1.15, 2.43)
Statins	1317	627/1317 (47.6)	1.03 (0.81, 1.31)	1.19 (0.84, 1.68)

Data are presented as numbers (%) and mean ± SD, or median [25th–75th percentile] if not normally distributed

^a^For quantitative variables, OR corresponds to an increase of 1 SD. Only BMI was natural log transformed before OR calculation

Ethnicity: EU (Europid), MENA (Middle East North Africa); AC (African or Caribbean), AS (Asian)

HBA_1c_ corresponds to the HBA_1c_ value determined in the 6 months prior to or in the first 7 days following hospital admission

Diabetic kidney disease defined as eGFR ≤60 ml min^−1^ [1.73 m]^−2^ and/or proteinuria

MRAs include spironolactone and eplerenone

ACS, acute coronary syndrome; CAR, coronary artery revascularisation; COPD, chronic obstructive pulmonary disease; GLP1-RA, glucagon-like peptide 1-receptor agonist; LLAR, lower limb artery revascularisation; NAFLD, non-alcoholic fatty liver disease; TIA, transient ischaemic attack

### Characteristics of COVID-19 on admission

Characteristics of COVID-19 on admission are provided in Table 2. The median duration of symptoms before admission was 5 days (25th–75th percentile, 2–8 days). As expected, the most common signs were fever (77.9%), cough (68.7%), fatigue (62.4%), dyspnoea (61.8%) and digestive disorders (34.5%). SARS-CoV-2 PCR testing was performed in 1268 participants, with a positive result in 96.8%. Thoracic CT imaging demonstrated typical ground-glass opacity and/or crazy paving in 818 individuals (90.0%). Biological findings were consistent with obvious infection as illustrated by a median C-reactive protein (CRP) at 77.8 (38.4–132.7) mg/l. Median plasma glucose at admission was 9.20 (6.80–12.62) mmol/l.

**Table 2:** COVID-19-related clinical, radiological and biological characteristics on admission in CORONADO participants, according to primary outcome (tracheal intubation and/or death within 7 days of admission), and death on day 7

Characteristic	Number of people with available data	All	Primary outcome (*n* = 382) OR (95% CI)	Death (*n* = 140) OR (95% CI)
COVID-19 symptoms	1313	1237/1313 (94.2)	3.20 (1.58, 6.49)	2.21 (0.79, 6.13)
Time between symptom onset and hospital admission (days)	1302	5 [2–8]	1.01 (0.99, 1.03)	0.96 (0.92, 0.99)
Clinical presentation				
Fever	1288	1003/1288 (77.9)	1.07 (0.80, 1.44)	0.73 (0.49, 1.10)
Fatigue	1239	773/1239 (62.4)	1.15 (0.89, 1.49)	1.13 (0.77, 1.65)
Cough	1270	872/1270 (68.7)	0.99 (0.76, 1.29)	0.87 (0.59, 1.28)
Cephalalgia	1193	157/1193 (13.2)	0.85 (0.58, 1.25)	0.44 (0.21, 0.92)
Dyspnoea	1292	798/1292 (61.8)	2.56 (1.95, 3.36)	2.29 (1.51, 3.47)
Rhinitis and/or pharyngeal symptoms	1178	111/1178 (9.4)	0.78 (0.49, 1.23)	0.39 (0.16, 0.99)
Ageusia and/or anosmia	1073	136/1073 (12.7)	0.73 (0.47, 1.12)	0.34 (0.14, 0.85)
Digestive disorders	1236	427/1236 (34.5)	0.83 (0.64, 1.08)	0.88 (0.60, 1.30)
Chest CT imaging				
Abnormal chest CT	896	844/896 (94.2)	1.16 (0.61, 2.21)	–
Ground-glass opacity/crazy paving	818	736/818 (90.0)	1.83 (1.02, 3.28)	1.70 (0.66, 4.32)
Biological findings				
Positive SARS-CoV-2 PCR	1268	1227/1268 (96.8)	2.44 (1.02, 5.85)	1.54 (0.47, 5.06)
Admission plasma glucose (mmol/l)^a^	940	9.20 [6.80–12.62]	1.28 (1.12, 1.48)	1.20 (0.98, 1.46)
Plasma creatinine (μmol/l)^a^	1196	91 [69–133]	1.24 (1.10, 1.40)	1.56 (1.33, 1.82)
eGFR (ml min^−1^ [1.73 m]^−2^)^a^	1196	69 [41.7–89.5]	0.82 (0.73, 0.93)	0.61 (0.52, 0.71)
ALT (%ULN)^a^	1068	0.62 [0.41–0.99]	1.25 (1.10, 1.42)	0.84 (0.67, 1.06)
AST (%ULN)^a^	1053	1.05 [0.75–1.51]	1.78 (1.54, 2.06)	1.34 (1.14, 1.59)
GGT (%ULN)^a^	983	0.94 [0.56–1.73]	1.25 (1.10, 1.43)	0.97 (0.78, 1.20)
Haemoglobin (g/l)^a^	1276	129 [114–143]	0.95 (0.84, 1.07)	0.96 (0.81, 1.14)
White cell count (10^3^/mm^3^)^a^	1269	6440 [4930–8610]	1.27 (1.12, 1.44)	1.43 (1.19, 1.70)
Lymphocyte count (10^3^/mm^3^)^a^	1211	990 [685–1400]	0.69 (0.60, 0.80)	0.75 (0.60, 0.92)
Platelet count (10^3^/mm^3^)^a^	1273	193 [151–246]	0.86 (0.76, 0.97)	0.86 (0.73, 1.02)
D-dimers (μg/l)^a^	397	830 [350–1571]	0.93 (0.76, 1.15)	1.25 (0.84, 1.86)
CRP (mg/l)^a^	1208	77.8 [38.4–132.7]	1.99 (1.69, 2.34)	1.49 (1.20, 1.84)
LDH (UI/l)^a^	566	351 [268–496]	2.43 (1.85, 3.18)	1.62 (1.10, 2.39)
CPK (UI/l)^a^	549	145 [72–319]	1.56 (1.30, 1.88)	1.68 (1.31, 2.17)
Fibrinogen (g/l)^a^	658	6.0 [4.8–7.2]	1.32 (1.09, 1.58)	1.05 (0.84, 1.31)

Data are presented as numbers (%) or median [25th–75th percentile] if not normally distributed

^a^All biological quantitative variables were natural log transformed. OR corresponds to an increase of 1 SD

eGFR was calculated according to the CKD-EPI formula

GGT, γ-glutamyl transferase; LDH, lactate dehydrogenase; ULN, upper limit of normal

Of interest, diabetes-related disorders were reported in 11.1% of the participants on admission with 132 episodes of severe hyperglycaemia, including 40 of ketosis, of which 19 were ketoacidosis, as well as 14 hypoglycaemic events, while severe anorexia was reported in 83 participants (6.3%).

### Factors prior to admission associated with study outcomes

In univariate analysis considering the primary outcome, male sex was more frequent (69.1% vs 63.2%, *p* = 0.0420) and BMI was significantly higher (median 29.1 [25.9–33.6] vs 28.1 [24.8–32.0] kg/m^2^, *p* = 0.0009) in patients who met the primary outcome compared with the others, as was the use of RAAS blockers (61.5% vs 55.3%, *p* = 0.0386) (Table 1 and electronic supplementary material [ESM] Table 1).

Furthermore, several characteristics prior to admission were associated with the risk of death on day 7 including age, hypertension, micro- and macrovascular diabetic complications and comorbidities such as heart failure or treated obstructive sleep apnoea (OSA). Among prior medications, metformin use was lower in people who died. In contrast, insulin therapy, RAAS blockers, β-blockers, loop diuretics and MRAs were found to be associated with death on day 7 (Table 1 and ESM Table 1).

**Fig. 2:**
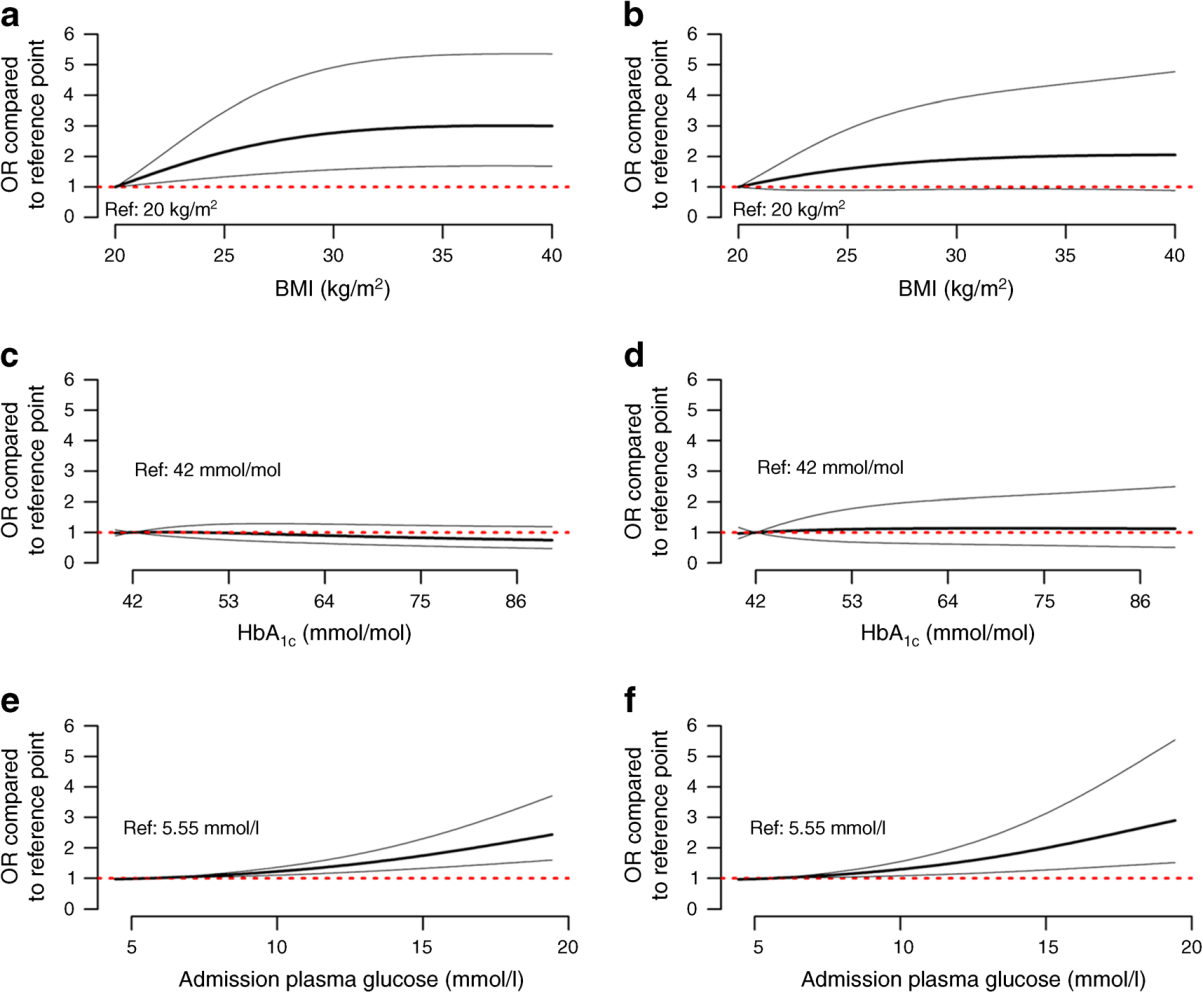
Sex- and age-adjusted ORs for the main outcome and for death, using logistic regression models with degree 2 multiple fractional polynomials. (**a**, **b**) OR for BMI for the primary outcome (**a**; *p* = 0.0001) and for death (**b**; *p* = 0.1488) on day 7 (reference value 20 kg/m^2^; *n* = 1117). (**c**, **d**) OR for HbA_1c_ for the primary outcome (**c**; *p* = 0.2897) and for death (**d**; *p* = 0.9129) on day 7 (reference value 42 mmol/mol; *n* = 846). (**e**, **f**) OR for admission plasma glucose for the primary outcome (**e**; *p* = 0.0001) and for death (**f**; *p* = 0.0059) on day 7 (reference value 5.55 mmol/l; *n* = 940). The thick black line gives the OR compared with the reference point, the thin grey lines are the 95% CI, and the red dotted red line (OR = 1) corresponds to a similar risk-level as the reference point

When using age- and sex-adjusted nonlinear models, BMI was significantly and positively associated with the primary outcome (*p* = 0.0001) but not with death on day 7 (*p* = 0.1488) (Fig. 2). In contrast, HbA_1c_ level was neither associated with the primary outcome nor with death on day 7.

Multivariable analyses were then conducted with characteristics prior to admission. BMI remained associated with the primary outcome in a model where sex and age were forced into models. When comorbidities and routine treatment were entered in an adjusted model with stepwise selection, BMI was the only independent factor associated with the primary outcome, with an adjusted OR of 1.28 (95% CI 1.10, 1.47) (Table 3). Finally, age, history of microvascular or macrovascular complications, and treated OSA were found to be independently associated with the risk of death on day 7 (Table 4). A sensitivity analysis conducted only in patients with a positive SARS-CoV-2 PCR test found similar results for both the primary outcome and death (data not shown).

**Table 3:** Multivariable analysis of the primary outcome in CORONADO participants: covariables prior to admission

	Model ‘prior to admission’: fully adjusted		Model ‘prior to admission’: stepwise selection with age and sex forced	
Patient characteristics	OR (95% CI)	*p* value	OR (95% CI)	*p* value
Age (+1 SD)	1.05 (0.90, 1.21)	0.5495	1.06 (0.92, 1.22)	0.4448
Sex (female/male)	0.76 (0.57, 1.03)	0.0777	0.75 (0.56, 1.01)	0.0559
BMI (+1 SD)	1.24 (1.06, 1.44)	0.0064	1.28 (1.10, 1.47)	0.0010
Treated OSA	1.15 (0.76, 1.73)	0.5036	–	–
ARBs and/or ACE inhibitors and/or MRAs	1.15 (0.86, 1.54)	0.3493	–	–

Models were applied in 1020 participants yielding 281 primary outcomes (27.5%)

BMI was natural log transformed. For quantitative variables, OR corresponds to an increase of 1 SD after standardisation

MRAs include spironolactone and eplerenone

**Table 4:** Multivariable analysis of the risk of death on day 7 in CORONADO participants: covariables prior to admission

	Model ‘prior to admission’: fully adjusted		Model ‘prior to admission’: stepwise selection with age and sex forced	
Patient characteristics	OR (95% CI)	*p* value	OR (95% CI)	*p* value
Age (+1 SD)	2.39 (1.67, 3.42)	<0.0001	2.48 (1.74, 3.53)	<0.0001
Sex (female/male)	0.78 (0.43, 1.40)	0.4023	0.78 (0.44, 1.38)	0.4007
Hypertension	0.76 (0.34, 1.70)	0.5087	–	–
Microvascular complications	1.78 (0.92, 3.44)	0.0846	2.14 (1.16, 3.94)	0.0153
Macrovascular complications	2.26 (1.25, 4.08)	0.0069	2.54 (1.44, 4.50)	0.0013
Heart failure	1.08 (0.54, 2.15)	0.8249	–	–
Active cancer	1.45 (0.77, 2.73)	0.2458	–	–
Treated OSA	2.65 (1.36, 5.19)	0.0044	2.80 (1.46, 5.38)	0.0020
β-Blockers	1.19 (0.69, 2.06)	0.5321	–	–
Metformin	0.80 (0.45, 1.43)	0.4532	–	–
Insulin	1.26 (0.72, 2.22)	0.4130	–	–
Loop diuretics	1.39 (0.76, 2.55)	0.2806	–	–
ARBs and/or ACE inhibitors and/or MRAs	1.22 (0.68, 2.20)	0.5069	–	–

Models were applied to 758 participants yielding 74 deaths (9.8%)

The OR for age corresponds to an increase of 1 SD after standardisation

MRAs include spironolactone and eplerenone

### Factors on admission associated with study outcomes

Regarding COVID-19 symptoms on admission, dyspnoea was positively associated with the primary outcome and with death on day 7, whereas cephalalgia, upper respiratory tract symptoms (rhinitis and/or pharyngeal symptoms), and ageusia/anosmia, as well as time between symptom onset and admission, were negatively associated with death on day 7 (Table 2 and ESM Table 2). Several biological parameters reflecting the severity of the infection were also associated with both primary outcome and death on day 7, such as CRP, creatine phosphokinase (CPK) and lymphopaenia. Reduced kidney function, assessed by admission eGFR, and increased plasma AST level, was associated with both outcomes (Table 2 and ESM Table 2). In age- and sex-adjusted nonlinear models, admission plasma glucose was significantly and positively associated with the primary outcome (*p* = 0.0001) and with death on day 7 (*p* = 0.0059) (Fig. 2).

The prognostic value of characteristics on admission was finally investigated in multivariable models (Table 5). Among clinical symptoms, dyspnoea was the only predictor of the primary outcome. Regarding biological parameters, lymphopaenia on admission was independently associated with the primary outcome, as were increased AST and CRP concentrations. Dyspnoea and plasma levels of both AST and CRP were also independently associated with the risk of death on day 7, as well as decrease in platelet count and eGFR (Table 6). Once again, similar results were obtained for both the primary outcome and death on day 7 when considering only the patients with a positive PCR test (data not shown).

**Table 5:** Multivariable analysis of the primary outcome in CORONADO participants: covariables on admission

	Model ‘on admission’: fully adjusted		Model ‘on admission’: stepwise selection with age and sex forced	
Patient characteristics	OR (95% CI)	*p* value	OR (95% CI)	*p* value
Age (+1 SD)	0.98 (0.77, 1.25)	0.8569	1.02 (0.81, 1.28)	0.8981
Sex (female/male)	1.46 (0.88, 2.41)	0.1410	1.44 (0.89, 2.32)	0.1417
BMI (+1 SD)	1.13 (0.89, 1.43)	0.3297	–	–
Dyspnoea	2.17 (1.34, 3.50)	0.0015	2.10 (1.31, 3.35)	0.0020
Admission plasma glucose (+1 SD)	1.14 (0.92, 1.42)	0.2391	–	–
eGFR (+1 SD)	0.81 (0.64, 1.01)	0.0643	–	–
AST (+1 SD)	2.19 (1.65, 2.90)	<0.0001	2.23 (1.70, 2.93)	<0.0001
Lymphocyte count (+1 SD)	0.70 (0.53, 0.94)	0.0161	0.67 (0.50, 0.88)	0.0050
Platelet count (+1 SD)	0.80 (0.63, 1.01)	0.0623	–	–
CRP (+1 SD)	2.00 (1.47, 2.73)	<0.0001	1.93 (1.43, 2.59)	<0.0001

Models were applied to 619 participants yielding 177 primary outcomes (28.6%)

For quantitative variables, OR corresponds to an increase of 1 SD after natural log transformation and standardisation, except for age, which was not natural log transformed

eGFR was calculated according to the CKD-EPI formula

**Table 6:** Multivariable analysis of the risk of death on day 7 in CORONADO participants: covariables on admission

	Model ‘clinical and biological’: fully adjusted		Model ‘clinical and biological’: stepwise selection with age and sex forced	
Patient characteristics	OR (95% CI)	*p* value	OR (95% CI)	*p* value
Age (+1 SD)	4.28 (2.64, 6.94)	<0.0001	4.12 (2.59, 6.55)	<0.0001
Sex (female/male)	0.92 (0.44, 1.92)	0.8237	1.02 (0.50, 2.09)	0.9523
Cephalalgia	1.55 (0.43, 5.58)	0.5029	–	–
Dyspnoea	2.73 (1.30, 5.73)	0.0079	2.80 (1.37, 5.72)	0.0049
Rhinitis and/or pharyngeal signs	0.46 (0.11, 1.96)	0.2957	–	–
Ageusia and/or anosmia	1.31 (0.43, 4.01)	0.6403	–	–
Admission plasma glucose (log, +1 SD)	1.30 (0.94, 1.82)	0.1148	–	–
eGFR (log, +1 SD)	0.51 (0.37, 0.69)	<0.0001	0.51 (0.38, 0.69)	<0.0001
AST (log, +1 SD)	1.93 (1.38, 2.71)	0.0001	1.85 (1.33, 2.56)	0.0003
White cell count (log, +1 SD)	1.29 (0.86, 1.92)	0.2126	–	–
Platelet count (log, +1 SD)	0.66 (0.47, 0.92)	0.0144	0.71 (0.53, 0.97)	0.0292
CRP (log, +1 SD)	1.70 (1.09, 2.66)	0.0202	1.87 (1.20, 2.89)	0.0052

Models were applied to 612 participants yielding 59 primary outcomes (9.6%)

For quantitative variables, OR corresponds to an increase of 1 SD after natural log transformation and standardisation, except for age, which was also standardised but not natural log transformed

eGFR was calculated according to the CKD-EPI formula

## Discussion

CORONADO is the first study specifically dedicated to people with diabetes infected with SARS-CoV-2 and admitted to hospital. CORONADO was designed to address three main goals: (1) assess the phenotypic characteristics of patients with diabetes hospitalised for COVID-19; (2) estimate the prevalence of the primary outcome, which combines death and tracheal intubation for mechanical ventilation within the first 7 days following admission; (3) identify in this specific population certain prognostic factors associated with early severity of COVID-19. When considering variables prior to admission, our results support no independent association between a severe course of COVID-19 and age, sex, long-term glucose control, chronic complications, hypertension or usual medications, including RAAS blockers and DPP-4 inhibitors. Only BMI turned out to be independently associated with the primary outcome. When considering variables on admission, dyspnoea, lymphopaenia, and increased AST and CRP levels were independent prognostic factors for severe course of COVID-19.

To our knowledge, CORONADO is the first study that provides precise information regarding the characteristics of diabetes in the severe forms of COVID-19. The study population roughly resembles the French population of people living with diabetes, except for HbA_1c_, which was clearly higher in our study (65 mmol/mol [8.1%]) compared with the nationwide ENTRED-2 survey participants older than 65 years (54 mmol/mol [7.1%]) [17]. Of note, there was no overrepresentation of declared type 1 diabetes (only 3.0% of participants) in people with diabetes hospitalised for COVID-19.

The primary outcome occurred in 29.0% of CORONADO participants. While the design of the present study did not enable comparison of the severity of COVID-19 in people with or without diabetes, 20.3% of the study population required tracheal intubation for mechanical ventilation with a mortality rate of 10.6% as early as 7 days after admission. The severity of the prognosis of COVID-19 observed in people with diabetes in the present study is in accordance with previous epidemiological studies [10–13, 18, 19], and meta-analyses [14, 20]. An important issue is the choice of our primary endpoint, which combines death (an unequivocal outcome) with tracheal intubation for mechanical ventilation. It should be emphasised that the latter outcome can result from different factors, which were impossible to standardise in all centres, such as (1) clinical deterioration, (2) refusal to be intubated, or (3) futility (i.e. a medical decision not to intubate), leading to potentially fewer patients actually intubated compared with those meeting intubation criteria.

Regarding the clinical characteristics of COVID-19 in CORONADO participants, there was a high prevalence of fever and respiratory symptoms (cough, dyspnoea) and, to a lesser extent, digestive disorders. In addition to symptoms directly related to COVID-19, people with diabetes can also require management of acute metabolic disorders. In particular, physicians should be warned not only of the risk of ketoacidosis but also of hypoglycaemia, probably favoured by COVID-19-induced anorexia without concomitant adaptation of glucose-lowering drugs.

With the aim of providing clinicians with criteria to evaluate the risk of severe COVID-19 on an individual level in people with diabetes, we performed multivariable analyses to identify pre-admission and on-admission prognostic factors. Since some preclinical studies previously highlighted potential mechanistic links between glucose control, immune response and MERS-CoV infection [21], we were particularly interested in studying the relationship between long-term glucose control and COVID-19 prognosis. In fact, we failed to find any association between HbA_1c_ (even with the highest values, >75 mmol/mol [9.0%]) and either the primary outcome or death on day 7. On the basis of this result and in order to increase the sample size for our analyses, we decided not to force HbA_1c_ in the multivariate models.

An interesting finding is the association of BMI with study outcomes. Indeed, in our study, BMI was positively and independently associated with the primary outcome, which is largely driven by tracheal intubation. Interestingly, a recent report on COVID-19 patients in ICU showed an association between BMI and the requirement for mechanical ventilation, irrespective of diabetic status [22]. However, such an association with BMI was no longer statistically significant when considering death on day 7. It should also be noted that the increased risk for the primary outcome appears to be less pronounced in patients with morbid obesity (grade 3, BMI ≥40 kg/m^2^) compared with those who were overweight or with grade 1–2 obesity, a situation previously described as the ‘obesity paradox’ in ICUs [23]. Additional studies are clearly warranted to decipher the link between obesity, metabolic complications and COVID-19 severity with specific attention to fat mass distribution, insulin resistance and inflammatory/immune profiles.

While hypertension was previously reported as the most prevalent comorbidity in the general population with severe COVID-19 [2, 9, 12], it was not independently associated with the severity of the disease in the study. In addition, RAAS blockers (ACE inhibitors, ARBs and MRAs) were not independently associated with the main outcome, supporting the recent recommendation not to discontinue RAAS blockade [24]. Moreover, we found no association between glucose-lowering drugs, including DPP-4 inhibitors, that have been suggested to potentially interfere with coronavirus infection and COVID-19 prognosis [21, 25].

Our complementary multivariable approach was suitable for the identification of characteristics on admission associated with COVID-19 prognosis, of particular relevance for the management of people with diabetes in the setting of an emergency room. Notably, we found an age- and sex-independent association between increased admission plasma glucose levels and the severity of COVID-19, as previously reported in critically ill patients [26]. However, we speculate that this observation is rather the consequence of the severity of the infection than a causal primary factor.

Another important result concerns the identification of the prognostic factors of early death in people with diabetes and COVID-19. Compared with the primary outcome, which reflects aggressive management in ICUs with tracheal intubation, death on day 7 was more prevalent in elderly participants with an OR >14 for people older than 75 years, compared with younger individuals. In addition, these individuals also very frequently exhibited complications of diabetes (microvascular and macrovascular complications, mainly coronary heart disease) as well as pulmonary diseases (such as OSA). As expected, they were also more frequently on insulin therapy and taking multiple drugs (such as diuretics). Conversely, metformin use was associated with a reduced risk of early death, probably reflecting a less advanced stage of diabetes with fewer comorbidities (such as severe chronic kidney disease) that contraindicate its use. In multivariable analyses, age, diabetic complications and treated OSA remained significantly and independently associated with death on day 7. In addition, dyspnoea, reduced eGFR and platelet count, and increased AST and CRP on admission were independent markers of early death.

The discrepancy between the primary combined outcome (mainly driven by tracheal intubation) and death on day 7 could be explained by the fact that there were medical decisions not to pursue aggressive therapy in this frail population. In contrast, our data can be considered reassuring for the majority of people living with type 1 diabetes. Indeed, there was no death in participants with type 1 diabetes younger than 65 years. Additional data collection is currently ongoing to provide a precise picture of the rare individuals with type 1 diabetes hospitalised for COVID-19.

Some limitations must be acknowledged in the current analysis. We focused on hospitalised COVID-19 cases and our results cannot be generalised to all people with COVID-19 and diabetes, especially those with a less severe form of the disease. A secondary limitation is the size of our study population and the large proportion (i.e. 35.7%) of patients without available HbA_1c_. This is in accordance with the observation that only 55% of the people with diabetes had had three or more HbA_1c_ determinations in the previous year according to French national registry data [27]. Finally, the present report focuses only on short-term prognosis (i.e. 7 days after admission) and one cannot exclude the possibility that diabetes characteristics prior to admission could be associated with severe COVID-19 outcomes in the longer term. However, strengths must be acknowledged such as the originality of the medical question leading to the CORONADO initiative and the inclusion of participants on a national basis. In addition, a large majority (>93%) of COVID-19 cases were confirmed with a positive PCR test, with few cases diagnosed from medical and/or radiological observations only. We also structured data collection in order to obtain a precise and standardised recording of phenotypic characteristics of the diabetic study population.

In conclusion, the CORONADO study refined the phenotypes of COVID-19 individuals with diabetes admitted to hospital and showed that chronic glycaemic control did not impact the immediate severity of COVID-19. Elderly populations with long-term diabetes with advanced diabetic complications and/or treated OSA were particularly at risk of early death, and might require specific management to avoid contamination with SARS-CoV-2. BMI also appears as an independent prognostic factor for COVID-19 severity in the population living with diabetes, requiring hospital admission.

## Electronic supplementary material

ESM 1 (PDF 405 kb)

## Data Availability

A data-sharing statement is available in the ESM.

## Abbreviations

ALT: Alanine aminotransferase
ARB: Angiotensin II receptor blocker
AST: Aspartate aminotransferase
CKD-EPI: Chronic Kidney Disease Epidemiology Collaboration
CORONADO: Coronavirus SARS-CoV-2 and Diabetes Outcomes
COVID-19: Coronavirus disease-2019
CPK: Creatine phosphokinase
CRP: C-reactive protein
CT: Computed tomography
DPP-4: Dipeptidyl peptidase 4
ICU: Intensive care unit
MERS-CoV: Middle East respiratory syndrome-related coronavirus
MRA: Mineralocorticoid-receptor antagonist
OSA: Obstructive sleep apnoea
RAAS: Renin–angiotensin–aldosterone system
SARS-CoV-2: Severe acute respiratory syndrome coronavirus-2
