# Supplementary material for: Phenotypic characteristics and prognosis of inpatients with COVID-19 and diabetes: the CORONADO study
Source: Diabetologia. 2020 May 29;63(8):1500–15. doi: 10.1007/s00125-020-05180-x (PMC7256180; doi:10.1007/s00125-020-05180-x)
Supplement: Supplementary file 1 — (PDF 405 kb) [file 125_2020_5180_MOESM1_ESM.pdf]

ESM Table 1. Clinical characteristics prior to admission of CORONADO participants, according to primary outcome (tracheal intubation and/or death) and death, on day 7

		Primary outcome at day 7			Death at day 7		
**Clinical features**	**Number of people with available data**	**No (N= 935)**	**Yes (N= 382)**	***P value***	**No (N = 1177)**	**Yes (N= 140)**	***P value***
**Sex (Female/Male)**	1317	344/935 (36.8%)	118/382 (30.9%)	0.0420	419/1177 (35.6%)	43/140 (30.7%)	0.2529
**Age (yrs)**	1317	69.7 ± 13.2	70 ± 12.5	0.6760	68.7 ± 12.9	79.4 ± 8.8	<0.0001
**Age class (yrs)**	1317			0.0520			<0.0001
< 55		106/935 (11.3%)	53/382 (13.9%)		156/1177 (13.3%)	3/140 (2.1%)	
55-64		206/935 (22.0%)	60/382 (15.7%)	0.0155	261/1177 (22.2%)	5/140 (3.6%)	0.9958
65-74		273/935 (29.2%)	121/382 (31.7%)	0.5479	371/1177 (31.5%)	23/140 (16.4%)	0.0595
≥75		350/935 (37.4%)	148/382 (38.7%)	0.3895	389/1177 (33.1%)	109/140 (77.9%)	<0.0001
**Type of diabetes**	1317			0.4910			0.0062
Type 2		828/935 (88.6%)	338/382 (88.5%)		1039/1177 (88.3%)	127/140 (90.7%)	
Type 1		30/935 (3.2%)	9/382 (2.4%)	0.4243	37/1177 (3.1%)	2/140 (1.4%)	0.2650
Other		46/935 (4.9%)	25/382 (6.5%)	0.2649	60/1177 (5.1%)	11/140 (7.9%)	0.2348
Diagnosed on admission		31/935 (3.3%)	10/382 (2.6%)	0.5239	41/1177 (3.5%)	0/140 (0%)	-
**Ethnicity**	1035			0.8029			0.0947
EU		459/741 (61.9%)	182/294 (61.9%)		567/925 (61.3%)	74/110 (67.3%)	
MENA		141/741 (19.0%)	55/294 (18.7%)	0.9281	176/925 (19.0%)	20/110 (18.2%)	0.6032
AC		126/741 (17.0%)	48/294 (16.3%)	0.8339	158/925 (17.1%)	16/110 (14.5%)	0.3817
AS		15/741 (2.0%)	9/294 (3.1%)	0.3361	24/925 (2.6%)	0/110 (0%)	-
**BMI (kg/m²)**	1117	28.1 [24.8; 32.0]	29.1 [25.9; 33.6]	0.0009	28.5 [25.1; 32.7]	27.7 [24.2; 32.5]	0.6179
**BMI class**	1117			0.0284			0.5592
< 25 kg/m²		215/797 (27.0%)	64/320 (20.0%)		247/1013 (24.4%)	32/104 (30.8%)	
25-29.9 kg/m²		294/797 (36.9%)	116/320 (36.2%)	0.1169	376/1013 (37.1%)	34/104 (32.7%)	0.1659
30-39.9 kg/m²		238/797 (29.9%)	121/320 (37.8%)	0.0031	327/1013 (32.3%)	32/104 (30.8%)	0.2876
≥40 kg/m²		50/797 (6.3%)	19/320 (5.9%)	0.4231	63/1013 (6.2%)	6/104 (5.8%)	0.5097
**Diabetes duration (yrs)**	772	13.6 ± 10.9	13.8 ± 10.7	0.8280	13.6 ± 10.8	13.9 ± 11.2	0.2193
**HbA_{1c} (mmol/mol)**	846	65.7 ± 21.3	64.8 ± 20.9	0.1853	65.5 ± 21.1	65.3 ± 21.5	0.8362
**HbA_{1c} (%)**	846	8.2 ± 1.9	8.1 ± 1.9	0.1853	8.1 ± 1.9	8.1 ± 2.0	0.8362
**HbA_{1c} (categories)**	846			0.6963			0.3143
< 53 mmol/mol (7 %)		180/641 (28.1%)	65/205 (31.7%)		227/777 (29.2%)	18/69 (26.1%)	
53-63 mmol/mol (7-7.9 %)		175/641 (27.3%)	53/205 (25.9%)	0.4096	203/777 (26.1%)	25/69 (36.2%)	0.174
64-74 mmol/mol (8-8.9 %)		123/641 (19.2%)	41/205 (20.0%)	0.7292	151/777 (19.4%)	13/69 (18.8%)	0.8281
≥ 75 mmol/mol (9 %)		163/641 (25.4%)	46/205 (22.4%)	0.2645	196/777 (25.2%)	13/69 (18.8%)	0.6355
**Hypertension**	1299	704/924 (76.2%)	299/375 (79.7%)	0.1682	886/1162 (76.2%)	117/137 (85.4%)	0.0171
**Dyslipidemia**	1255	454/899 (50.5%)	186/356 (52.2%)	0.5769	567/1123 (50.5%)	73/132 (55.3%)	0.2959
**Tobacco use**	1029			0.2049			0.9249
Never		431/742 (58.1%)	151/287 (52.6%)		530/936 (56.6%)	52/93 (55.9%)	
Former		274/742 (36.9%)	116/287 (40.4%)	0.1937	355/936 (37.9%)	35/93 (37.6%)	0.9831
Current		37/742 (5.0%)	20/287 (7.0%)	0.1391	51/936 (5.4%)	6/93 (6.5%)	0.6901
**Long-term diabetes complications**							
**Microvascular complications**	883	297/657 (45.2%)	116/226 (51.3%)	0.1120	345/798 (43.2%)	68/85 (80.0%)	<0.0001
Severe diabetic retinopathy	954	46/701 (6.6%)	20/253 (7.9%)	0.4711	55/864 (6.4%)	11/90 (12.2%)	0.0409
Diabetic kidney disease	1066	256/773 (33.1%)	99/293 (33.8%)	0.8357	297/967 (30.7%)	58/99 (58.6%)	<0.0001
History of Diabetic Foot Ulcer	1232	60/887 (6.8%)	16/345 (4.6%)	0.1661	65/1106 (5.9%)	11/126 (8.7%)	0.2104
**Macrovascular complications**	1189	338/852 (39.7%)	147/337 (43.6%)	0.2120	399/1063 (37.5%)	86/126 (68.3%)	<0.0001
Ischemic heart disease (ACS/CAR)	1251	237/890 (26.6%)	99/361 (27.4%)	0.7739	274/1117 (24.5%)	62/134 (46.3%)	<0.0001
Cerebrovascular disease (stroke or IAT)	1267	116/907 (12.8%)	47/360 (13.1%)	0.8985	133/1134 (11.7%)	30/133 (22.6%)	0.0005
Peripheral artery disease (Major amputation/LLAR)	1285	106/917 (11.6%)	39/368 (10.6%)	0.6225	120/1151 (10.4%)	25/134 (18.7%)	0.005
**Comorbidities**							
Heart failure	1206	106/861 (12.3%)	34/345 (9.9%)	0.2298	114/1083 (10.5%)	26/123 (21.1%)	0.0007
NAFLD or liver cirrhosis	1107	82/805 (10.2%)	37/302 (12.3%)	0.3238	110/994 (11.1%)	9/113 (8.0%)	0.3155
Active Cancer	1282	136/915 (14.9%)	58/367 (15.8%)	0.6711	166/1147 (14.5%)	28/135 (20.7%)	0.0561
COPD	1278	96/913 (10.5%)	37/365 (10.1%)	0.8417	115/1142 (10.1%)	18/136 (13.2%)	0.2548
Treated OSA	1189	94/857 (11.0%)	50/332 (15.1%)	0.0533	120/1061 (11.3%)	24/128 (18.8%)	0.0160
Organ graft	1302	26/925 (2.8%)	12/377 (3.2%)	0.7176	36/1164 (3.1%)	2/138 (1.4%)	0.2899
End stage renal failure	831	48/608 (7.9%)	12/223 (5.4%)	0.2176	55/728 (7.6%)	5/103 (4.9%)	0.3256

**Routine treatment before admission**							
Metformin	1317	533/935 (57.0%)	213/382 (55.8%)	0.6788	683/1177 (58.0%)	63/140 (45.0%)	0.0035
Sulfonylurea/glinides	1317	259/935 (27.7%)	108/382 (28.3%)	0.8337	335/1177 (28.5%)	32/140 (22.9%)	0.1632
DPP4-inhibitors	1317	202/935 (21.6%)	83/382 (21.7%)	0.9606	258/1177 (21.9%)	27/140 (19.3%)	0.4746
GLP1-RA	1317	80/935 (8.6%)	43/382 (11.3%)	0.1276	114/1177 (9.7%)	9/140 (6.4%)	0.2141
Insulin	1317	357/935 (38.2%)	147/382 (38.5%)	0.9191	434/1177 (36.9%)	70/140 (50.0%)	0.0027
Loop diuretics	1317	175/935 (18.7%)	77/382 (20.2%)	0.5465	204/1177 (17.3%)	48/140 (34.3%)	<0.0001
Thiazide diuretics	1317	186/935 (19.9%)	81/382 (21.2%)	0.5913	239/1177 (20.3%)	28/140 (20.0%)	0.9322
Potassium-sparing diuretics	1317	40/935 (4.3%)	19/382 (5.0%)	0.5800	49/1177 (4.2%)	10/140 (7.1%)	0.1116
MRA	1317	38/935 (4.1%)	15/382 (3.9%)	0.9083	43/1177 (3.7%)	10/140 (7.1%)	0.0514
Beta blockers	1317	312/935 (33.4%)	130/382 (34.0%)	0.8173	377/1177 (32.0%)	65/140 (46.4%)	0.0007
ACE inhibitors	1317	243/935 (26.0%)	111/382 (29.1%)	0.2546	307/1177 (26.1%)	47/140 (33.6%)	0.0599
ARBs	1317	265/935 (28.3%)	124/382 (32.5%)	0.1374	344/1177 (29.2%)	45/140 (32.1%)	0.4749
ARBs and/or ACE inhibitors	1317	505/935 (54.0%)	232/382 (60.7%)	0.0259	645/1177 (54.8%)	92/140 (65.7%)	0.0146
ARBs and/or ACE inhibitors and/or MRA	1317	517/935 (55.3%)	235/382 (61.5%)	0.0386	657/1177 (55.8%)	95/140 (67.9%)	0.0070
Statins	1317	443/935 (47.4%)	184/382 (48.2%)	0.7950	555/1177 (47.2%)	72/140 (51.4%)	0.3388

Data are presented as numbers (%) and mean ± SD, or median (25th; 75th percentile) if not normally distributed.
P values are calculated using Wald test (univariate logistic regression).
Ethnicity: EU (Europid), MENA (Middle East North Africa); AC (African or Caribbean), AS (Asian); Glycated A1c corresponds to the glycated hemoglobin determined in the 6 months prior to or in the first 7 days following hospital admission; GLP1-RA, Glucagon-like Peptide-Receptor Agonist; ACS, acute coronary syndrome; CAR, coronary artery revascularization ; IAT, ischemic transient accident; LLAR, lower limb artery revascularization; COPD, chronic obstructive pulmonary disease; OSA, obstructive sleep apnea. DKD, defined as eGFR 60 mL/min/1.73 m^2 or lower and/or proteinuria; NAFLD, non-alcoholic fatty liver disease; DPP4, Dipeptidyl peptidase 4; GLP-1RA, Glucagon-Like Peptide 1-Receptor Agonist; diuretics; ACE Inhibitors, angiotensin converting enzyme-inhibitors; ARB, angiotensin-2 receptor blocker; MRA, mineralocorticoid-receptor antagonist (i.e. spironolactone and eplerenone)

ESM Table 2. COVID-19-related clinical, radiological and biological characteristics on admission in CORONADO participants, according to primary outcome (tracheal intubation and/or death) and death, on day 7

		**Primary outcome at day 7**			**Death at day 7**		
	**Number of persons with available data**	**No (N= 935)**	**Yes (N= 382)**	***P value***	**No (N = 1177)**	**Yes (N= 140)**	***P value***
**COVID-19 symptoms**	1313	865/932 (92.8%)	372/381 (97.6%)	0.0012	1102/1174 (93.9%)	135/139 (97.1%)	0.1296
**Time between symptom onset and hospital admission (days)**	1302	5 [2; 8]	5 [2; 8]	0.5319	5 [2; 8]	3 [1; 6]	0.0119
**Clinical presentation**							
Fever	1288	711/917 (77.5%)	292/371 (78.7%)	0.6467	904/1152 (78.5%)	99/136 (72.8%)	0.1326
Fatigue	1239	545/887 (61.4%)	228/352 (64.8%)	0.2754	690/1111 (62.1%)	83/128 (64.8%)	0.5450
Cough	1270	628/914 (68.7%)	244/356 (68.5%)	0.9533	787/1141 (69.0%)	85/129 (65.9%)	0.4745
Cephalalgia	1193	118/864 (13.7%)	39/329 (11.9%)	0.4107	149/1072 (13.9%)	8/121 (6.6%)	0.0285
Dyspnea	1292	513/919 (55.8%)	285/373 (76.4%)	<0.0001	692/1155 (59.9%)	106/137 (77.4%)	0.0001
Rhinitis and/or pharyngeal signs	1178	85/851 (10.0%)	26/327 (8.0%)	0.2849	106/1059 (10.0%)	5/119 (4.2%)	0.0469
Agueusia and/or Anosmia	1073	106/781 (13.6%)	30/292 (10.3%)	0.1496	131/973 (13.5%)	5/100 (5.0%)	0.0206
Digestive disorders	1236	317/887 (35.7%)	110/349 (31.5%)	0.1605	386/1108 (34.8%)	41/128 (32%)	0.5274
**Chest CT imaging**							
Abnormal chest CT	896	609/648 (94.0%)	235/248 (94.8%)	0.6567	763/815 (93.6%)	81/81 (100%)	-
Ground-glass opacity/crazy paving	818	522/589 (88.6%)	214/229 (93.4%)	0.0416	663/740 (89.6%)	73/78 (93.6%)	0.2690
**Biological findings**							
Positive SARS-CoV-2 PCR	1268	865/900 (96.1%)	362/368 (98.4%)	0.0455	1094/1132 (96.6%)	133/136 (97.8%)	0.4767
Admission plasma glucose (mmol/l)	940	8.80 [6.70; 12.10]	10.30 [7.10; 14.53]	0.0005	9.00 [6.80; 12.44]	10.40 [7.16; 13.90]	0.0775
Plasma creatinine (µmol/l)	1196	88.0 [67.0; 125]	100 [73.5; 150]	0.0005	89.0 [67.6; 126]	123 [86.5; 177]	<0.0001
eGFR (CKD-EPI) (ml/min/1.73 m²)	1196	72.0 [44.6; 90.6]	59.4 [36.9; 85.5]	0.0016	72.0 [44.9; 91.5]	43.1 [29.7; 69.0]	<0.0001
ALT (%ULN)	1068	0.58 [0.40; 0.94]	0.71 [0.46; 1.10]	0.0007	0.62 [0.42; 1.00]	0.58 [0.38; 0.81]	0.1354
AST (%ULN)	1053	0.97 [0.68; 1.34]	1.32 [0.94; 1.96]	<0.0001	1.03 [0.74; 1.46]	1.27 [0.93; 1.86]	0.0005
GGT (%ULN)	983	0.92 [0.55; 1.60]	1.11 [0.62; 2.07]	0.0009	0.95 [0.56; 1.77]	0.83 [0.51; 1.50]	0.7801
Hemoglobin (g/dl)	1276	12.9 [11.5; 14.3]	12.8 [11.3; 14.4]	0.3924	12.9 [11.4; 14.3]	12.7 [11.1; 14.3]	0.6311
White cell count ($10^3/mm^3$)	1269	6320 [4870; 8400]	6650 [5118; 9400]	0.0001	6400 [4890; 8462]	7380 [5530; 10100]	0.0001
Lymphocyte count ($10^3/mm^3$)	1211	1030 [700; 1492]	820 [595; 1200]	<0.0001	1000 [700; 1408]	880 [500; 1150]	0.0075
Platelet count ($10^3/mm^3$)	1273	198 [156; 249]	182 [142; 233]	0.0134	194 [152; 246]	176 [136; 238]	0.0860
D-dimers (µg/l)	397	820 [364; 1570]	850 [330; 1732]	0.5155	809 [337; 1568]	1130 [639; 1746]	0.2742
C reactive protein, (mg/l)	1208	66 [31.1; 119]	110.4 [68.8; 179.5]	<0.0001	75 [36; 127]	113 [54; 179.3]	0.0002
LDH (UI/l)	566	323 [249; 425]	440 [334; 638]	<0.0001	342 [265; 474]	451 [352; 638]	0.0142
CPK (UI/l)	549	122 [64; 265]	204 [100; 455]	<0.0001	134 [69; 295]	253 [150; 522]	<0.0001
Fibrinogen (g/l)	658	5.8 [4.7; 7.0]	6.3 [5.2; 7.4]	0.0037	6 [4.8; 7.2]	6.2 [5.0; 6.8]	0.6810

Data are presented as numbers (%) and mean ± SD, or median (25th; 75th percentile) if not normally distributed. P values are calculated using Wald test (univariate logistic regression).

eGFR, estimated glomerular filtration rate, according to the CKD-EPI formula; ALT, alanine aminotransferase; AST, aspartate amino-transferase; GGT, gamma-glutamyl transferase; LDH, lactate dehydrogenase; CPK, creatine phosphokinase; CRP, C reactive protein; ULN, Upper limit of normal;

Scientific committee (City, Scientific domain):

S Hadjadj, (Nantes, Diabetology) Chairman; B Cariou, (Nantes, Diabetology) Principal Investigator, National Coordinator; B Bauduceau (Paris, Diabetology Gerontology) on behalf of FFRD; D Boutoille (Nantes, Infectiology); C Chaumeil (Paris, patients association) on behalf of Fédération Française des Diabétiques; JF Gautier (Paris, Diabetology); P Gourdy (Toulouse, Diabetology); V Kerlan (Brest, Diabetology), on behalf of SFE; B Laviolle (Rennes, Epidemiology); F Cazenave-Roblot (Poitiers, Infectiology), on behalf of SPILF; M Pichelin (Nantes, Study manager); R Robert (Poitiers, Intensive Care); R Roussel (Paris, Diabetology); JF Thebaut (Paris, patients association) on behalf of Fédération Française des Diabétiques; C Thivolet (Lyon, Diabetology), on behalf of SFD; M Wargny (Nantes, Methodology).

List of collaborators

MAHOT Pascale, CHU de Nantes, Nantes, France, pascale.moreau@chu-nantes.fr

HADJADJ Samy, CHU de Nantes, Nantes, France, samy.hadjadj@univ-nantes.fr

FOURNIER-GUILLOUX Anne-Laure, CHU de Nantes, Nantes, France, annelaure.fournierguilloux@chu-nantes.fr

MAUDUIT Nicolas, CHU de Nantes, Nantes, France, nicolas.mauduit@chu-nantes.fr

BIGOT- CORBEL Edith, CHU de Nantes, Nantes, France, edith.bigot@chu-nantes.fr

BOUREAU Anne-Sophie, CHU de Nantes, Nantes, France, annesophie.boureau@chu-nantes.fr

DE DECKER Laure, CHU de Nantes, Nantes, France, laure.dedecker@chu-nantes.fr

ERNOULD Audrey, CHU de Nantes, Nantes, France, audrey.ernould@chu-nantes.fr

PRIMOT Claire, CHU de Nantes, Nantes, France, claire.primot@chu-nantes.fr

SEGUIN Anne, CHU de Nantes, Nantes, France, anne.seguin@chu-nantes.fr

JOLIVEAU Marielle, CHU de Nantes, Nantes, France, marielle.joliveau@chu-nantes.fr

POUVREAU Sonia, CHU de Nantes, Nantes, France, sonia.pouvreau@chu-nantes.fr

FOURNIER Chloé, CHU de Nantes, Nantes, France, chloe.fournier@chu-nantes.fr

THUREAU Jeremy, CHU de Nantes, Nantes, France, jeremy.thureau@chu-nantes.fr

FONTENEAU Edith, CHU de Nantes, Nantes, France, edith.fonteneau@chu-nantes.fr

HUBLAIN Pamela, CHU de Nantes, Nantes, France, Pamela.HUBLAIN@chu-nantes.fr

AGASSE Carole, CHU de Nantes, Nantes, France, Carole.AGASSE@chu-nantes.fr

DE KERGARADEC Mathilde, CHU de Nantes, Nantes, France, laurence.dekergaradec@chu-nantes.fr

MINVILLE Vincent, CHU Toulouse, Toulouse, France, minville.v@chu-toulouse.fr

VARDON-BOUNES Fanny, CHU Toulouse, Toulouse, France, bounes.f@chu-toulouse.fr

MARTIN-BLONDEL Guillaume, CHU Toulouse, Toulouse, France, martin-blondel.g@chu-toulouse.fr

TURNIN Marie-Christine, CHU Toulouse, Toulouse, France, turnin.mc@chu-toulouse.fr

HANAIRE Hélène, CHU Toulouse, Toulouse, France, hanaire.h@chu-toulouse.fr

MANSUY Jean-Michel, CHU Toulouse, Toulouse, France, mansuy.jm@chu-toulouse.fr

FABRE Didier, CHU Toulouse, Toulouse, France, fabre.d@chu-toulouse.fr

ARHAINX Marie-Blanche, CHU Toulouse, Toulouse, France, arhainx.mb@chu-toulouse.fr

CAZALS Laurent, CHU Toulouse, Toulouse, France, cazals.l@chu-toulouse.fr

COMBES Laure, CHU Toulouse, Toulouse, France, combes.l@chu-toulouse.fr

LAMI Emmanuelle, CHU Toulouse, Toulouse, France, lami.e@chu-toulouse.fr

CIANFERANI Mallory, CHU Toulouse, Toulouse, France, cianferani.m@chu-toulouse.fr

MEGARBANE Bruno, Hôpital LARIBOISIERE, Paris, France, bruno.megarbane@aphp.fr

LEROY Pierre, Hôpital LARIBOISIERE, Paris, France, pierre.leroy@aphp.fr

VIDAL-TRECAN Tiphaine, Hôpital LARIBOISIERE, Paris, France, tiphaine.vidal-trecan@aphp.fr

LAPLANCHE Jean-Louis, Hôpital LARIBOISIERE, Paris, France, jean-louis.laplanche@aphp.fr

MOULY Stéphane, Hôpital LARIBOISIERE, Paris, France, stephane.mouly@aphp.fr

TAHER Malak, Hôpital BICHAT, Paris, France, malak.taher@aphp.fr

ABOULEKA Yawa, Hôpital BICHAT, Paris, France, yawa.abouleka@aphp.fr

YAKER Fetta, Hôpital BICHA, Paris, France, fettaamel.yaker@aphp.fr

CARLIER Aurelie, Hôpital BICHAT, Paris, France, aurelie.carlier@aphp.fr

BOUTTEN Anne, Hôpital BICHAT, Paris, France, anne.boutten@aphp.fr

HALLOT-FERON Marilyne, Hôpital BICHAT, Paris, France, marilyne.feron@aphp.fr

MOURAH Fadila, Hôpital BICHAT, Paris, France, fadila.mourah@gmail.com

BLOND Emilie, Hôpital Lyon Sud, Pierre Bénite, France, emilie.blond@chu-lyon.fr

ROLLAND Muriel, Hôpital Lyon Sud, Pierre Bénite, France, muriel.rolland@chu-lyon.fr

VERDECHO MENDEZ Josep, Hôpital Lyon Sud, Pierre Bénite, France, josep.verdecho-mendez@chu-lyon.fr

ALEXANDRE Marine, Hôpital Lyon Sud, Pierre Bénite, France, marine.alexandre@chu-lyon.fr

POTTECHER Julien, Nouvel hôpital civil, Strasbourg, France, julien.pottecher@chru-strasbourg,fr

RICHER Emilie, Nouvel hôpital civil, Strasbourg, France, emilie.richerdupont@chru-strasbourg.fr

LUCA Florina, Hôpital Hautepierre, Strasbourg, France, florina.luca@chru-strasbourg,fr

LESSINGER Jean-Marc, Nouvel hôpital civil, Strasbourg, France, Jean-Marc.LESSINGER@chru-strasbourg.fr

BAHOUGNE Thibault, Hospices civils, Strasbourg, France, thibault.bahougne@chru-strasbourg,fr

GUERCI Bruno, CHU Brabois Université de Lorraine, Nancy, France, b.guerci@chru-nancy.fr

LUDWIG Lisa, CHU Brabois Université de Lorraine, Nancy, France, L.LUDWIG@chru-nancy.fr

BENZIRAR Siham, CHU Brabois Université de Lorraine, Nancy, France, s.benzirar@chru-nancy.fr

MALAPLATE Catherine, CHU Brabois Université de Lorraine, Nancy, France, c.malaplate@chu-nancy.fr

MATTON Thierry, CHU Brabois Université de Lorraine, Nancy, France, t.matton@chru-nancy.fr

POISSY Julien, Hôpital salengro CHU, Lille, France, julien.poissy@chru-lille.fr

FAURE Karine, Hôpital Huriez CHRU, Lille, France, karine.faure@chru-lille.fr

FONTAINE Pierre, Hôpital Huriez CHRU, Lille, France, pierre.fontaine@chru-lille.fr

VAMBERGUE Anne, Hôpital Huriez CHRU, Lille, France, anne.vambergue@chru-lille.fr

PEKAR Jean David, Hôpital Huriez CHRU, Lille, France, jeandavid.PEKAR@chru-lille.fr

LAMBERT Marc, Hôpital Calmette CHU, Lille, France, marc.lambert@chru-lille.fr

YELNIK Cécile, Hôpital Calmette CHU, Lille, France, cecile.yelnik@chru-lille.fr

BRUANDET Amélie, Hôpital Huriez CHRU, Lille, France, amelie.bruandet@chru-lille.fr

PETIT Laurent, CHU de Bordeaux, Bordeaux, France, laurent.petit@chu-bordeaux.fr

NEAU Didier, CHU de Bordeaux, Bordeaux, France, didier.neau@chu-bordeaux.fr

RIGALLEAU Vincent, CHU de Bordeaux, Pessac, France, vincent.rigalleau@chu-bordeaux.fr

BERARD Annie, CHU de Bordeaux, Bordeaux, France, annie.berard@chu-Bordeaux.fr

GALIOOT Amandine, CHU de Bordeaux, Pessac, France, amandine.galioot@chu-bordeaux.fr

COUDROY Remy, CHU Poitiers, Poitiers, France, Remi.COUDROY@chu-poitiers.fr

THILLE Arnaud, CHU Poitiers, Poitiers, France, arnaud.thille@chu-poitiers.fr

RAMMAERT Blandine, CHU Poitiers, Poitiers, France, blandine.rammaert@chu-poitiers.fr

PIGUEL Xavier, CHU Poitiers, Poitiers, France, xavier.piguel@chu-poitiers.fr

BENHENDA Nesrine, CHU Poitiers, Poitiers, France, Nesrine.BENHENDA@chu-poitiers.fr

HUSSON Camille, CHU Poitiers, Poitiers, France, Camille.HUSSON@chu-poitiers.fr

OLIVIER Celine, CHU Poitiers, Poitiers, France, celine.olivier@chu-poitiers.fr

TORREMOCHA Florence, CHU Poitiers, Poitiers, France, florence.torremocha@chu-poitiers.fr

FRATY Mathilde, CHU Poitiers, Poitiers, France, mathilde.fraty@chu-poitiers.fr

FLAMEN D'ASSIGNY Marie, CHU Poitiers, Poitiers, France, marie.flamen-dassigny@chu-poitiers.fr

MIOT Aurelie, CHU Poitiers, Poitiers, France, aurelie.miot@chu-poitiers.fr

BOSSARD Valentin, CHU Poitiers, Poitiers, France, valentin.bossard988@gmail.com

KLOUCHE Kada, Hôpital Lapeyronie, Chu Montpellier, France, k-klouche@chu-montpellier.fr

MAKINSON Alain, Hôpital Lapeyronie, Chu Montpellier, France, a-makinson@chu-montpellier.fr

BONNET Jean-Baptiste, Hôpital Lapeyronie, Chu Montpellier, France, jean-baptiste-bonnet@chu-montpellier.fr

FOULONGNE Vincent, Hôpital St Eloi, Chu Montpellier, France, v-foulongne@chu-montpellier.fr

GALTIER Florence, Hôpital St Eloi, Chu Montpellier, France, f-galtier@chu-montpellier.fr

AUBRON Cécile, CHU de Brest, Brest, France, cecile.aubron@ch-brest.fr

ANSART Séverine, CHU de Brest, Brest, France, severine.ansart@chu-brest.fr

QUINIOU Pascale, CHU de Brest, Brest, France, pascale.quiniou@chu-brest;fr

CARRE Jean- Luc, CHU de Brest, Brest, France, jean-luc.carre@chu-brest.fr

QUESNOT Stéphane, CHU de Brest, Brest, France, stephane.quesnot@chu-brest.fr

SCHWEBEL Carole, CHU Grenoble Alpes, Grenoble, France, Cschwebel@chu-grenoble.fr

EPAULARD Olivier, CHU Grenoble Alpes, Grenoble, France, OEpaulard@chu-grenoble.fr

BENHAMOU Pierre-Yves, CHU Grenoble Alpes, Grenoble, France, PYBenhamou@chu-grenoble.fr

BETRY Cécile, CHU Grenoble Alpes, Grenoble, France, Cbetry@chu-grenoble.fr

BOREL Anne-Laure, CHU Grenoble Alpes, Grenoble, France, ALBorel@chu-grenoble.fr

LABLANCHE Sandrine, CHU Grenoble Alpes, Grenoble, France, Slablanche@chu-grenoble.fr

GUERGOUR Dorra, CHU Grenoble Alpes, Grenoble, France, Dguergour@chu-grenoble.fr

DUCLOS Catherine, Hôpital AVICENNE, Bobigny, France, catherine.duclos@aphp.fr

GUYOT Erwan, Hôpital AVICENNE, Bobigny, France, erwan.guyot@aphp.fr

DENIAU Aurore, Hôpital AVICENNE, Bobigny, France, aurore.deniau@aphp.fr

NGUYEN Phucthutrang, Hôpital AVICENNE, Bobigny, France, phucthutrang.nguyen@aphp.fr

REZNIK Yves, CHU Caen Normandie, Caen, France, reznik-y@chu-caen.fr

ALLOUCHE Stéphane, CHU Caen Normandie, Caen, France, allouche-s@chu-caen.fr

GUITTET Lydia, CHU Caen Normandie, Caen, France, guittet-l@chu-caen.fr

GRANGE Steven, CHU Rouen, Rouen, France, steven.grange@chu-rouen.fr

ETIENNE Manuel, CHU Rouen, Rouen, France, manuel.etienne@chu-rouen.fr

BRUNEL Valéry, CHU Rouen, Rouen, France, valery.brunel@chu-rouen.fr

LAGIER Jean-Christophe, IHU Marseille, Marseille, France, JeanChristophe.LAGIER@ap-hm.fr

RAOULT Didier, IHU Marseille, Marseille, France, didier.raoult@ap-hm.fr

DUTOUR Anne, CHU Nord et Conception, Marseille, France, anne.dutour@ap-hm.fr

BOULLLU Sandrine, CHU Nord, Marseille, France, sandrine.boullu@ap-hm.fr

CERINO Mathieu, CHU Conception, Marseille, France, mathieu.cerino@ap-hm.fr

ROMAIN Fanny, CHU Conception, Marseille, France, fanny.romain@ap-hm.fr

HOUSSAYS Marie, CHU Conception, Marseille, France, marie.houssays@ap-hm.fr

QUENOT Jean Pierre, CHU François Mitterand, Dijon, France, jean-pierre.quenot@chu-dijon.fr

PIROTH Lionel, CHU François Mitterand, Dijon, France, lionel.piroth@chu-dijon.fr

VERGÈS Bruno, CHU François Mitterand, Dijon, France, bruno.verges@chu-dijon.fr

DUVILLARD Laurence, CHU François Mitterand, Dijon, France, laurence.duvillard@chu-dijon.fr

BONNOTTE Bernard, CHU François Mitterand, Dijon, France, bernard.bonnotte@chu-dijon.fr

MERCAT Alain, CHU ANGERS, Angers, France, almercat@chu-angers.fr

DUBEE Vincent, CHU ANGERS, Angers, France, vincent.dubee@chu-angers.fr

RODIEN Patrice, CHU ANGERS, Angers, France, parodien@chu-angers.fr

REYNIER Pascal, CHU ANGERS, Angers, France, pareynier@chu-angers.fr

LARCHER Françoise, CHU ANGERS, Angers, France, frjoubaud@chu-angers.fr

JOUBAUD Françoise, CHU ANGERS, Angers, France, frjoubaud@chu-angers.fr

ANDREU Marie-Rita, CHU ANGERS, Angers, France, marierita.andreu@chu-angers.fr

URBANSKI Geoffrey, CHU ANGERS, Angers, France, geoffrey.urbanski@chu-angers.fr

HUBERT Laurent, CHU ANGERS, Angers, France, lahubert@chu-angers.fr

ANNWEILER Cedric, CHU ANGERS, Angers, France, ceannweiler@chu-angers.fr

DELLAMONICA Jean, CHU de Nice - Hôpital de l'Archet, Nice, France, dellamonica.j@chu-nice.fr

COURJON Johan, CHU de Nice - Hôpital de l'Archet, Nice, France, courjon.j@chu-nice.fr

CHEVALIER Nicolas, CHU de Nice - Hôpital de l'Archet, Nice, France, chevalier.n@chu-nice.fr

CHINETTI Giulia, CHU de Nice - Hôpital Pasteur, Nice, France, chinetti.g@chu-nice.fr

CHAFAI Magda, CHU de Nice - Hôpital de l'Archet, Nice, France, chafai.m@chu-nice.fr

MOURVILLIER Bruno, CHU de Reims, Reims, France, bmourvillier@chu-reims.fr

BANI-SADR Firouze, CHU de Reims, Reims, France, fbanisadr@chu-reims.fr

DELEMER Brigitte, CHU de Reims, Reims, France, bdelemer@chu-reims.fr

GILLERY Philippe, CHU de Reims, Reims, France, pgillery@chu-reims.fr

LABEDADE Pascale, Centre Hospitalier Sud-Francilien, Corbeil-Essonnes, France, pascale.labedade@chsf.fr

CHABROL Amélie, Centre Hospitalier Sud-Francilien, Corbeil-Essonnes, France, amelie.chabrol@chsf.fr

PENFORNIS Alfred, Centre Hospitalier Sud-Francilien, Corbeil-Essones, France, alfred.penfornis@chsf.fr

PETIT Catherine, Centre Hospitalier Sud-Francilien, Corbeil-Essones, France, catherine.petit@chsf.fr

ADLER Maxime, Centre Hospitalier Sud-Francilien, Corbeil-Essonnes, France, maxime.adler@chsf.fr

DUBOST Clément, HIA Bégin, Saint Mandé, France, clement.dubost@intradef.gouv.fr

CONAN Pierre-Louis, HIA Bégin, Saint Mandé, France, pierre-louis.conan@intradef.gouv.fr

BORDIER Lyse, HIA Bégin, Saint Mandé, France, lyse.bordier@intradef.gouv.fr

CEPPA Franck, HIA Bégin, Saint Mandé, France, franck.ceppa@intradef.gouv.fr

GARCIA Cyril, HIA Bégin, Saint Mandé, France, cyril1.garcia@intradef.gouv.fr

SOLLIER Mathilde, HIA Bégin, Saint Mandé, France, mathilde.sollier@intradef.gouv.fr

DUPUY Olivier, GH Paris Saint Joseph, Paris, France, odupuy@hpsj.fr

LAPLANCE Sophie, GH Paris Saint Joseph, Paris, France, slaplanche@hpsj.fr

BILLUART Olivier, GH Paris Saint Joseph, Paris, France, obilluart@hpsj.fr

AROULANDA Marie Joseph, GH Paris Saint Joseph, Paris, France, mjaroulanda@hpsj.fr

OLIVIER Frédérique, CH CAHORS, Cahors, France, frederique.olivier@ch-cahors.fr

AYON Florence, CH CAHORS, Cahors, France, florence.ayon@ch-cahors.fr

WILHELM Nathalie, CH CAHORS, Cahors, France, nathalie.wilhelm@ch-cahors.fr

EPELBOIN Loic, CHU Cayennes, Cayenne, France, loic.epelboin@ch-cayenne.fr

SABBAH Nadia, CHU Cayennes, Cayenne, France, nadia.sabbah@ch-cayenne.fr

CHARPIN Aurelie, CHU Cayennes, Cayenne, France, aurelie.charpin@ch-cayenne.fr

SQUARA Pierre, Clinique Ambroise Paré, Paris, France, pierre.squara@orange.fr

BELLIARD Olivier, Clinique Ambroise Paré, Paris, France, olivier_belliard@yahoo.fr

DUBOIS Claude, Clinique Ambroise Paré, Paris, France, claude.dubois@clinique-a-pare.fr

MARRE Michel, Clinique Ambroise Paré, Paris, France, marre.michel@gmail.com

AUCHABIE Johann, CH Cholet, Cholet, France, johann.auchabie@ch-cholet.fr

COURTOIS Roxane, CH Cholet, Cholet, France, roxane.courtois@ch-cholet.fr

DURIEZ Thierry, CH Cholet, Cholet, France, thierry.duriez@ch-cholet.fr

MERGEY Tiphaine, CH Cholet, Cholet, France, tiphaine.mergey@ch-cholet.fr

VALLEE Laura, CH Cholet, Cholet, France, laura.vallee@ch-cholet.fr

SEGUIN Laetitia, CH Cholet, Cholet, France, laetitia.seguin@ch-cholet.fr

LANOIX Jean-Philippe, CHU Amiens- Picardie, Amiens, France, lanoix.jean-philippe@chu-amiens.fr

SORIOT-THOMAS Sandrine, CHU Amiens-Picardie, Amiens, France, soriot-thomas.sandrine@chu-amiens.fr

BOURGEOIS-DESCOULS Anne-Marie, CHU Amiens-Picardie, Amiens, France, bourgeois.anne-marie@chu-amiens.fr

DESAILLOUD Rachel, CHU Amiens-Picardie, Amiens, France, dessaillud.rachelàchu-amiens.fr

GERMAIN Natacha, CHU de Saint Etienne, Saint Etienne, France, natacha.germain@chu-st-etienne.fr

GALUSCA Bogdan, CHU de Saint Etienne, Saint Etienne, France, bogdan.galusca@chu-st-etienne.fr

BELLETON Gwenaelle, CHU de Saint Etienne, Saint Etienne, France, gwenaelle.belleton@chu-st-etienne.fr

MAROUANI Nesrine, CHU de Saint Etienne, Saint Etienne, France, nesrine.marouani@chu-st-etienne.fr

PALAGHIU Delia, CHU de Saint Etienne, Saint Etienne, France, delia.palaghiu@chu-st-etienne.fr

HAMMOUR Amira, CHU de Saint-Etienne, Saint-Etienne, France, amira.hammour@chu-st-etienne.fr

BERDAGUER Fernando, Hôpital Nord Franche-Comté, Belfort, France, fberdaguer@hotmail.com

WINISZEWSKI Patrice, Hôpital Nord Franche-Comté, Belfort, France, patrice.winiszewski@hnfc.fr

KLOPFENSTEIN Thimothée, Hôpital Nord Franche-Comté, Belfort, France, Timothee.KLOPFENSTEIN@hnfc.fr

ZAYET Hajer, Hôpital Nord Franche-Comté, Belfort, France, Hajer.ZAYET@hnfc.fr

WINISZEWSKI Patrice, Hôpital Nord Franche-Comté, Belfort, France, Patrice.WINISZEWSKI@hnfc.fr

ZANUSSO Marie, Hôpital Nord Franche-Comté, Belfort, France, marie.zanusso@hnfc.fr

GARNIER Pauline, Hôpital Nord Franche-Comté, Belfort, France, pauline.garnier@hnfc.fr

JULIER Ingrid, CH de Ales, Ales, France, dr.julier@ch-ales.fr

HAMZAOUI Karim, CH de Ales, Ales, France, dr.hamzaoui@ch-ales.fr

MARTY-GRES Sophie, CH de Ales, Ales, France, biologie@ch-ales.fr

EL SADKI Tarik, CH de Ales, Ales, France, biologie@ch-ales.fr

CADOT Lucile, CH de Ales, Ales, France, biologie@ch-ales.fr

DUBOST Jean-Louis, CH de Pontoise, Pontoise, France, jean-louis.dubost@ght-novo.fr

GONFROY Céline, CH de Pontoise, Pontoise, France, celine.gonfroy@ght-novo.fr

CAMPINOS Catherine, CH de Pontoise, Pontoise, France, catherine.campinos@ght-novo.fr

MARTRES Pascale, CH de Pontoise, Pontoise, France, pascale.martres@ght-novo.fr

COULHON Marie Pierre, CH de Pontoise, Pontoise, France, marie-pierre.coulhon@ght-novo.fr

FLAUS FURMANIUK Anna, CHU Felix Guyon, Saint Denis, France, anna.flaus-furmaniuk@chu-reunion.fr

ALLOU Nicolas, CHU Felix Guyon, Saint Denis, France, nicolas.allou@chu-reunion.fr

BACHIR Marwa, CHU Felix Guyon, Saint Denis, France, marwa.bachir@chu-reunion.fr

HOANG Stella, CHU Felix Guyon, Saint Denis, France, stella.hoang@chu-reunion.fr

KEMBELLEC Candice, CHU Felix Guyon, Saint Denis, France, candice.kembellec@chu-reunion.fr

SUPLY Olivia, CHU Felix Guyon, Saint Denis, France, olivia.suply@chu-reunion.fr

KHARCHA Fatima, CHU Felix Guyon, Saint Denis, France, fatima.kharcha@chu-reunion.fr

DEVOUGE Anne-Claire, CHU Felix Guyon, Saint Denis, France, anne-claire.devouge@chu-reunion.fr

FLAUS-FURMANUK Anna, CHU Felix Guyon, Saint Denis, France, anna.flaus-furmaniuk@chu-reunion.fr

BASTARD Sophie, CHU Felix Guyon, Saint Denis, France, sophie.bastard@chu-reunion.fr

RAFFRAY Loic, CHU Felix Guyon, Saint Denis, France, loic.raffray@chu-reunion.fr

RENOU Frederic, CHU Felix Guyon, Saint Denis, France, frederic.renou@chu-reunion.fr

BOJARSKI Aude, CHU Felix Guyon, Saint Denis, France, aude.bojarski@chu-reunion.fr

PAUL Caroline, CHU Felix Guyon, Saint Denis, France, caroline.paul@chu-reunion.fr

BORSU Karine, CHU Felix Guyon, Saint Denis, France, karine.borsu@chu-reunion.fr

GORLIN Angelique, CHU Felix Guyon, Saint Denis, France, angelique.gorlin@chu-reunion.fr

DI BERNARDO Servane, CHU Felix Guyon, Saint Denis, France, servane.dibernardo@chu-reunion.fr

TRUONG VAN UT Carole, CHU Felix Guyon, Saint Denis, France, carole.truong-van-ut@chu-reunion.fr

RENAUD Stephane, CHU Felix Guyon, Saint Denis, France, stephane.renaud@chu-reunion.fr

VIGNOLES Antoine, CHU Felix Guyon, Saint Denis, France, antoine.vignoles@chu-reunion.fr

FOCH Emilie, CHU Felix Guyon, Saint Denis, France, emilie.foch@chu-reunion.fr

MASSE Laurie, CHU Felix Guyon, Saint Denis, France, laurie.masse@chu-reunion.fr

GRAND Hubert, robert boulin, Libourne, France, hubert.grand@ch-libourne.fr

FERRAND Helene, robert boulin, Libourne, France, helene.ferrand@ch-libourne.fr

RAFFAITIN-CARDIN Christelle, robert boulin, Libourne, France, christelle.raffaitin@ch-libourne.fr

ZELLAGUI Hadjer, robert boulin, Libourne, France, hadjer.zellagui@ch-libourne.fr

CASTANG-BRACHET Celine, robert boulin, Libourne, France, celine.castang@ch-libourne.fr

BOURY Frederique, robert boulin, Libourne, France, frederique.boury@ch-libourne.fr

ALVAREZ TENA Ana, CH Albi, Albi, France, ana.alvareztena@ch-albi,fr

MOURA Isabelle, CH Albi, Albi, France, isabelle.moura@ch-albi.fr

KALFON Pierre, LOUIS PASTEUR, Le Coudray, France, pkalfon@ch-chartres.fr

DARASTEANU Juliana, LOUIS PASTEUR, Le Coudray, France, jdarasteanu@ch-cahrtres.fr

MONIER Arnaud, LOUIS PASTEUR, Le Coudray, France, amonier@ch-chartres.fr

FOUCAULT Pascal, LOUIS PASTEUR, Le Coudray, France, pfoucault@ch-chartres.fr

DEPUILLE Alexandra, LOUIS PASTEUR, Le Coudray, France, adepuille@ch-chartres.fr

LAUGIER-ROBIOLLE Stéphanie, CH D'AUCH, 32000, France, s.laugier-robiolle@ch-auch.fr

CANEIRO Patrick, CH D'AUCH, 32000, France, p.caneiro@ch-auch.fr

BASSO Maud, CH D'AUCH, 32000, France, maud.basso@ch-auch.fr

LARGER Etienne, Hôpital COCHIN, Paris, France, etienne.larger@aphp.fr

BENZENATI Wahiba, Hôpital COCHIN, Paris, France, wahiba.benzenati-ext@aphp.fr

AIT BACHIR Leila, Hopital francobritannique, Levallois, France, leila.aitbachir@ihfb.org

CUSSAC PILLEGAND Camille, Hopital francobritannique, Levallois, France, camille.cussac-pillegand@ihfb.org

VASSE Marc, Hopital francobritannique, Suresnes, France, marc.vasse@hopital-foch.fr

MICHARD Christophe, CH du Forez, Montbrison, France, christophe.michard@ch-forez.fr

MONTANIER Nathanaëlle, CH du Forez, Montbrison, France, nathanaelle.montanier@ch-forez.fr

MILLOT Luc, CH du Forez, Montbrison, France, luc.millot@ch-forez.fr

CREPET Françoise, CH du Forez, Montbrison, France, francoise.crepet@ch-forez.fr

RATSIMBA Danielle, CH du Forez, Montbrison, France, danielle.ratsimba@ch-forez.fr

BOUILLER Kevin, CHU JEAN MINJOZ, Besancon, France, kbouiller@chu-besancon.fr

BRUCKERT Isabelle, CHU JEAN MINJOZ, Besancon, France, ibruckert@chu-besancon.fr

CLERGEOT Annie, CHU JEAN MINJOZ, Besancon, France, aclergeot@chu-besancon.fr

SCHILLO Franck, CHU JEAN MINJOZ, Besancon, France, fschillo@chu-besancon.fr

VIGNES Dorothée, CHU Antoine Béclère, Clamart, France, dorothée.vignes@aphp.fr

LACHGAR Hamoud, CHU Antoine Béclère, Clamart, France, hamoud.lachgar@aphp.fr

LAMBERT DE CURSAY Claire, CHU Antoine Béclère, Clamart, France, claire.lambertdecursay@aphp.fr

LEVANTE Stéphane, CHU Antoine Béclère, Clamart, France, stephane.levante@aphp.fr

AUREGAN Jean Charles, CHU Antoine Béclère, Clamart, France, jean-charles.auregan@aphp.fr

MERLET Antoine, CH Bretagne Atlantique, Vannes, France, antoine.merlet@ch-bretagne-atlantique.fr

ZARAGOZA Cécile, CH Bretagne Atlantique, Vannes, France, cecile.zaragora@ch-bretagne-atlantique.fr

ARNAULT Gwénaëlle, CH Bretagne Atlantique, Vannes, France, gwenaelle.arnault@ch-bretagne-atlantique.fr

LE LOUPP Anne-Gaëlle, CH Bretagne Atlantique, Vannes, France, anne-gaelle-le8loupp@ch-bretagne-atlantique.fr

LESIEUR Olivier, Saint Louis, La Rochelle, France, olivier.lesieur@ch-larochelle.fr

RONCATO-SABERAN Mariam, Saint Louis, La Rochelle, France, mariam.roncato@ch-larochelle.fr

GOUET Didier, Saint Louis, La Rochelle, France, didier.gouet@ch-larochelle.fr

LEMARIE Romain, Saint Louis, La Rochelle, France, romain.lemarie@ch-larochelle.fr

ALLANO Hong_An, Saint Louis, La Rochelle, France, hong-an.allano@ght-atlantique17.fr

VIVIER Emmanuel, Saint-Joseph Saint-Luc, Lyon, France, evivier@ch-stjoseph-stluc-lyon.fr

PARISET Caroline, Saint-Joseph Saint-Luc, Lyon, France, cpariset@ch-stjoseph-stluc-lyon.fr

LUYTON Cédric, Saint-Joseph Saint-Luc, Lyon, France, cluyton@ch-stjoseph-stluc-lyon.fr

PECQUET Mathieu, Saint-Joseph Saint-Luc, Lyon, France, mpecquet@ch-stjoseph-stluc-lyon.fr

PERARD Laurent, Saint-Joseph Saint-Luc, Lyon, France, lperard@ch-stjoseph-stluc-lyon.fr

VUILLERMOZ-BLAS Sylvie, Saint-Joseph Saint-Luc, Lyon, France, svuillermoz@ch-stjoseph-stluc-lyon.fr

KACKI Nicolas, CHD de Vendée, La Roche Sur Yon, France, nicolas.kacki@chd-vendée.fr

CHARRIER Patricia, CHD de Vendée, La Roche Sur Yon, France, patricia.charrier@chd-vendée.fr

DESROYS DU ROURE Françoise, CHD de Vendée, La Roche Sur Yon, France, francois.desroysduroure@chd-vendee.fr

BONNEFONT-ROUSSELOT Dominique, Pitié-Salpêtrière, Paris, France, dominique.rousselot@aphp.fr

LAROCHE Suzanne, Pitié-Salpêtrière, Paris, France, Suzanne.laroche@aphp.fr

PHAN Franck, Pitié-Salpêtrière, Paris, France, franck.phan@aphp.fr

HARTEMANN Agnès, Pitié-Salpêtrière, Paris, France, agnes.hartemann@aphp.fr

CAUSSY Cyrielle, CHU LYON SUD, Pierre Benite, France, cyrielle.caussy@chu-lyon.fr

BLOND Emilie, CHU LYON SUD, Pierre Benite, France, emilie.blond@chu-lyon.fr

GUERIN Claude, Hôpital Croix Rousse, Lyon, France, claude.guerin@chu-lyon.fr

PERPOINT Thomas, Hôpital Croix Rousse, Lyon, France, thomas.perpoint@chu-lyon.fr

MOULIN Philippe, Hôpital Louis pradel, Lyon, France, philippe.moulin@chu-lyon.fr

CARTIER Régine, Hôpital Louis pradel, Lyon, France, regine.cartier@chu-lyon.fr

HARIRI Geoffroy, Hôpital Saint-Antoine, Paris, France, geoffroy.hariri@aphp.fr

CHOPIN Dorothée, Hôpital Saint-Antoine, Paris, France, dorothee.chopin@aphp.fr

BOURCIGAUX Nathalie, Hôpital Saint-Antoine, Paris, France, nathalie.bourcigaux@aphp.fr

CHAIGNEAU Emmanuelle, Hôpital Saint-Antoine, Paris, France, emmanuelle.chaigneau@aphp.fr

CHRISTIN-MAITRE Sophie, Hôpital Saint-Antoine, Paris, France, sophie.christin-maitre@aphp.fr

DONADILLE Bruno, Hôpital Saint-Antoine, Paris, France, bruno.donadille@aphp.fr

FEVE Bruno, Hôpital Saint-Antoine, Paris, France, bruno.feve@aphp.fr

LAMOTHE Sophie, Hôpital Saint-Antoine, Paris, France, sophie.lamothe@aphp.fr

SARFATI Julie, Hôpital Saint-Antoine, Paris, France, julie.sarfati@aphp.fr

PERNET Pascal, Hôpital Saint-Antoine, Paris, France, pascal.pernet@aphp.fr

CHAMBON Anne, CH Côtes Basques, Bayonne, France, achambon@ch-cotebasque.fr

DEMARSY Delphine, CH Côtes Basques, Bayonne, France, ddemarsy@ch-cotebasque.fr

CAMPAGNE Hugo, CH Côtes Basques, Bayonne, France, hcampagne@ch-cotebasque.fr

LATIL-PLAT Françoise, CH Avignon, Avignon, France, fplat@ch-avignon.fr

BERNE Monica, CH Avignon, Avignon, France, MBeyrne@ch-avignon.fr

GRINAND Marilyne, CH Avignon, Avignon, France, GRINAND.Marilyne@ch-avignon.fr

TOUZET Marion, CH Avignon, Avignon, France, TOUZET.Marion@ch-avignon.fr

ZABULON Aydrey, CHU Martinique, Fort de France, France, audrey.zabulon@chu-martinique.fr

CRASPAG Jocelyne, CHU Martinique, Fort de France, France, jocelyne.craspag@chu-martinique.fr

LEDOUX Catherine, CHU Martinique, Fort de France, France, catherine.ledoux@chu-martinique.fr

CONTARET Cedric, CHU Martinique, Fort de France, France, Cedric.CONTARET@chu-martinique.fr

JANAND-DELENNE Blandine, CH du Pays d'Aix, Aix en Provence, France, bdelenne@ch-aix.fr

GIRAUD Anaïs, CH du Pays d'Aix, Aix en Provence, France, agiraud@ch-aix.fr

LACRIMINI Marie Lou, CH du Pays d'Aix, Aix en Provence, France, mlacrimini@ch-aix.fr

ARRIVIE Joëlle, CH de Bigorre, Tarbes, France, jarrivie@ch-tarbes-vic.fr

ANCELLE Deborah, CH Le Havre, Le Havre, France, deborah.ancelle@ch-havre.fr

GUILLOIS Carine, CH Le Havre, Le Havre, France, carine.guillois@ch-havre.fr

FREMY Bénédicte, CH Agen, Agen, France, fremyb@ch-agen-nerac.fr

CHAALAL Amina, CH Agen, Agen, France, chaalalam@ch-agen-nerac.fr

BARRANDE Gaëlle, CH Argenteuil, Argenteuil, France, gaelle.barrande@ch-argenteuil.fr

DORANGE Anne, CH Le Mans, Le Mans, France, adorange@ch-lemans.fr

ROUANET Eglantine, CH Le Mans, Le Mans, France, erouanet@ch-lemans.fr

SERET-BEGUE Dominique, CH Gonesse, Gonesse, France, dominique.seret-begue@ch-gonesse.fr

SAOUD Audrey, CH Gonesse, Gonesse, France, audrey.saoud@ch-gonesse.fr

GUEDJ Anne-Marie, CH Nîmes, Nîmes, France, anne.marie.guedj@chu-nimes.fr

BEDOS Nathalie, CH Nîmes, Nîmes, France, nathalie.bedos@chu-nimes.fr

VELAYOUDOM Fritz-Line, CHU Guadeloupe, Les Abymes, France, fritz-line.velayoudom@univ-antilles.fr

DUMAS Marie, Hôpital St Vincent de Paul, Lille, France, dumas.marie@ghicl.net

GONDA Benoite, Hôpital St Vincent de Paul, Lille, France, Gonda.Benoite@ghicl.net

COFFIN Christine, CH Perigueux, Perigueux, France, christine.coffin@ch-perigueux.fr

GIBIAT Stéphanie, CH Perigueux, Perigueux, France, urc@ch-perigueux.fr

LUNGO Myriam, CH de Bastia, Bastia, France, myriam.lungo@gmail.com

BULLY Chantal, Les Portes du Sud, Venissieux, France, C.BULLY@LESPORTESDUSUD.NET

SERUSCLAT Pierre, Les Portes du Sud, Venissieux, France, p.serusclat@lesportesdusud.net

BULLY Stella, Les Portes du Sud, Venissieux, France, stellabully.ecsel@gmail.com

CARRE Patricia, Les Portes du Sud, Venissieux, France, patcarre69@gmail.com

LEBERRE Jean-Philippe, Medipôle Hôpital Mutualiste, Villeurbanne, France, j.leberre@resamut.fr

ELKHOURY Carlos, Medipôle Hôpital Mutualiste, Villeurbanne, France, c.elkhoury@resamut.fr

THIEUX Marine, Medipôle Hôpital Mutualiste, Villeurbanne, France, m.thieux@resamut.fr

PARADISI-PRIEUR Laetitia, Medipôle Hôpital Mutualiste, Villeurbanne, France, l.paradisi-prieur@resamut.fr

DATA SHARING STATEMENT

What data will be made available (deidentified participant data, participant data with identifiers, data dictionary, or other specified data set):
No sharing of participant data is allowed by our regulatory authorities. So far, French regulations have not validated deidentified data or avatar for data sharing. Our statement might be modified in case French law changes.

Whether additional, related documents will be available (eg, study protocol, statistical analysis plan, informed consent form)
We will be happy to share study protocol, SAP and information document.

• When these data will be available (beginning and end date, or "with publication", as applicable)
Study protocol, SAP and information document will be made available with publication.
Data dictionary will be made available Summer 2020 (JULY 15th)

• Where the data will be made available (including complete URLs or email addresses if relevant);
The CORONADO website is not active yet but we will give access to the scientific committee through our website, as soon as it is launched.
Direct requests can be directed to PI (bertrand.cariou@univ-nantes.fr) or Chairman of the scientific committee (samy.hadjadj@univ-nantes.fr)

• By what access criteria data will be shared (including with whom, for what types of analyses, by what mechanism – eg, with or without investigator support, after approval of a proposal, with a signed data access agreement - or any additional restrictions).
Our data-base is open for any collaborative work with priority to academic partnership. Any proposal for collaboration requires examination by the scientific committee and the sponsor (CHU Nantes). A structured application proposal for collaboration will be available on request.
